# Global, regional, and national burden of hip dislocation, 1990–2021, and Bayesian age-period-cohort predictions: Systematic analysis of the Global Burden of Disease study, 2021

**DOI:** 10.1371/journal.pone.0340294

**Published:** 2026-01-15

**Authors:** Shun Lin, Xiu Yang, Baorong Cai, Jie Xiao, Qiping Wu, Guanyuan Yi, Xuesong Han, Feng Shen

**Affiliations:** 1 Fuzong Clinical Medical College of Fujian Medical University, FuZhou, China; 2 900th Hospital of PLA Joint Logistic Support Force, FuZhou, China; Atal Bihari Vajpayee Institute of Medical Sciences & Dr Ram Manohar Lohia Hospital, INDIA

## Abstract

**Background:**

Hip dislocation is a severe, clinically rare trauma. The global incidence, trends, and causes of hip dislocation have not been precisely documented. We aimed to explore the burden of hip dislocation from 1990 to 2021 and predict the trends over the next two decades.

**Methods:**

Data from the Global Burden of Disease (GBD) Study 2021 were used to describe the incidence, years lived with disability (YLD), and the corresponding age-standardized rates (ASRs) of hip dislocation for 1990 and 2021, stratified by sex, age, and the Socio-Demographic Index (SDI) across 21 GBD regions and 204 countries and territories. Joinpoint regression analysis was employed to investigate the temporal trends in incidence. Correlations between ASRs and SDI at the regional and national levels were assessed using Pearson correlation analysis. A Bayesian Age-Period-Cohort (BAPC) model was constructed to predict the disease burden for hip dislocation for the next 20 years.

**Results:**

In 2021, 2,429,935 new cases (95% uncertainty interval [UI] 1,634,456–3,549,251) of hip dislocation were recorded globally, with 109,146 YLDs (95% UI 57,181–190,161) attributable to hip dislocation. Between 1990 and 2021, the incidence of hip dislocation increased by 18.4% (95% UI 8.5–30.5%) and the YLDs increased by 57.2% (95% UI 50.9–66.4%). The corresponding average annual percentage changes (AAPC) in the age-standardized incidence rate and YLD were −0.712 (95% UI −0.79 to −0.634) and −0.535 (95% UI −0.59 to −0.481), respectively. The highest disease burden occurred in regions with high-SDI. Yemen and Afghanistan had the highest age-standardized incidence rates (ASIRs). At both regional and national levels, statistically significant non-linear relationships were observed between the ASRs of hip dislocation and SDI. The highest incidence occurred in young adulthood for males and old age for females. The leading cause was falls. The BAPC model predicts an increasing burden of hip dislocation among females over the next 20 years.

**Conclusion:**

The global burden of hip dislocation has increased over the last three decades, and is relatively larger in some high-SDI regions and low-income countries. The burden of hip dislocation is expected to increase, particularly among elderly females, due to population aging. Therefore‌, appropriate preventive measures should be developed to mitigate this trend.

## Introduction

Hip dislocation is a clinically rare, serious traumatic injury [1] that causes severe pain and loss of motion and may also lead to a variety of serious sequelae, including post-traumatic hip osteoarthritis [2], ischemic necrosis of the femoral head [3], recurrent dislocation [4] and sciatic nerve injury [5].The presence of concomitant injuries may increase the complexity of treatment, prolong hospitalization, and affect long-term prognosis, and thus place a significant burden on the patient [6–9]. Early identification and appropriate management are crucial for this type of injury, as these fundamental principles help optimize treatment outcomes, reduce the risk of long-term complications, and decrease the likelihood of medical malpractice allegations [10].

There is a lack of published research on the global incidence of hip dislocation. Previous studies only reported simple descriptive statistics, focused on certain causes of injury, had small sample sizes, or were only conducted in one country or region. Young and middle-aged males are generally accepted to be at higher risk of hip dislocation than females of the same age and males in other age groups [11]. This may be related to the fact that this group is more frequently involved in high-risk activities, such as strenuous sports [12] and driving motor vehicles [13]. Furthermore, patients with congenital hip dysplasia, individuals who have undergone hip replacement surgery, and obese individuals have higher risks of hip dislocation [14,15]. The increasing annual incidence of traumatic hip dislocation in North America has prompted concern about the potential of this traumatic injury to become a significant threat to public health and well-being [1]. However, despite considerable efforts by numerous researchers, the incidence of hip dislocation worldwide remains inadequately quantified due to current research limitations, regional imbalances in medical resources, inconsistent diagnostic criteria, and challenges related to data collection. Furthermore, there has been no comprehensive analysis of the global trends in hip dislocation.

The Global Burden of Disease Study 2021 [GBD 2021] was coordinated by the Institute for Health Metrics and Evaluation in the United States and aimed to establish comprehensive estimates of the burden and risk factors for a variety of diseases and injuries [16]. By utilizing data on hip dislocation from the GBD 2021, we aimed to comprehensively assess the global burden, trends, etiology, and factors that influence hip dislocation in order to provide data to support decision making and enable improved resource allocation in public health, optimization of public health policies, and long-term health planning.

## Methods

### Data source

Data on hip dislocation were obtained from the GBD 2021 (https://vizhub.healthdata.org/gbd-results/). The GBD 2021 assessed the global burden of a total of 371 diseases, impairments, and injuries (including 88 risk factors) across 204 countries and territories in the period from 1990 to 2021 [17]. We extracted the annual number of incident cases, years lived with disability (YLD), age-standardized incidence rates (ASIR), and age-standardized YLD rates (ASYR) for hip dislocation from 1990 to 2021. All data are categorized by gender, age, region, and country and include the corresponding 95% uncertainty intervals (UI).

The authors affirm that the ethical policies of the journal, as outlined on the journal’s author guidelines page, have been strictly adhered to. As this research was based on publicly accessible data, ethical approval was not necessary.

### Definitions

The case definition for hip dislocation relied on ICD-10 code S73.0 and ICD-9 codes 835.0 to 835.13. As an indicator of quality of life, YLD was calculated as the number of healthy years of life forfeited due to disability related to hip dislocation. Age-standardized rate (ASR) were calculated per 100,000 individuals based on the age distribution of the corresponding standard population in the GBD 2021.

The Socio-Demographic Index (SDI) is derived from the geometric mean of the total fertility rate, per capita income, and the average years of education, and ranges from 0 to 1; higher SDI reflects higher socio-economic status. The 204 countries and territories in the GBD 2021 dataset were stratified into five distinct categories based on the SDI: low SDI (0–0.25), lower-middle SDI (0.25–0.50), middle SDI (0.50–0.75), upper-middle SDI (0.75–0.90), and high SDI (0.90–1.00). These categories are consistent with the official classification used by the GBD 2021 study [17].

### Statistical analysis

The temporal trends in the disease burden of hip dislocation from 1990 to 2021 were assessed via Joinpoint regression analysis using Joinpoint Regression Software (Version 5.1.0, National Cancer Institute, Rockville, MD, USA). This model takes the natural logarithm of the ASR across different time periods, and then calculates the annual percentage change (APC) and 95% confidence interval (CI) for each time period.

The average annual percentage change (AAPC) in the ASRs from 1990 to 2021 were also determined. The lower bound of the 95% CI exceeding zero indicates a significant upward trend in the ASR; conversely, the upper bound of the 95% CI falling below zero indicates a downward trend [18]. In addition, Pearson’s correlation analysis was used to assess the strength and direction of the correlations between the ASRs and the SDI at both the regional and national levels.

To predict the disease burden of hip dislocation over the next 20 years, we utilized the Bayesian Age-Period-Cohort (BAPC) model [19]. The BAPC analysis was conducted using the BAPC package for model specification and INLA for Bayesian computation. Data processing was performed using the tidyverse package and data.table to optimize the large dataset. The model validation steps included posterior predictive checks and convergence diagnostics.

All other statistical analyses and data visualizations were performed using R (version 4.4.0). P-values less than 0.05 were considered statistically significant.

All analyses were conducted between July and October 2024 using the most recent GBD 2021 data release to ensure temporal consistency with reference population estimates.

## Results

### Global overview

In 2021, the global incidence of hip dislocation was estimated at 2,429,935 cases (95% UI 1,634,456–3,549,251; Table 1) and the YLDs reached 109,146 (95% UI 57,181–190,161; S1 Table in S1 File). Between 1990 and 2021, the incidence of hip dislocation increased by 18.4% (95% UI 8.5–30.5%) and YLDs increased by 57.2% (95% UI 50.9–66.4%).

**Table 1:** Incident cases and ASIR of hip dislocation in 1990 and 2021, and the AAPC values for 1990 to 2021, stratified by global and SDI regions.

Characteristic	1990		2021		1990–2021	
	Incident cases	ASIR per 100,000	Incident cases	ASIR per 100,000	Change of number	AAPC
	n (95% UI)	n (95% UI)	n (95% UI)	n (95% UI)	n (95% UI)	n (95% CI)
Global	2052925 (1388083,2841633)	38.29 (26.02,53.53)	2429935 (1634456,3549251)	30.63 (20.65,44.8)	18.4% (8.5%,30.5%)	-0.712 (-0.79 - -0.634)
SDI regions						
High SDI	345744 (226932,488618)	38.77 (25.5,54.54)	376455 (244135,552492)	31.83 (21.08,46.72)	8.9% (-2.2%,20.8%)	-0.657 (-0.828 - -0.484)
High-middle SDI	485816 (318447,698272)	45.24 (29.86,65.13)	475619 (309052,725238)	35.97 (23.6,54.33)	-2.1% (-11.6%,8.7%)	-0.816 (-0.969 - -0.663)
Middle SDI	612512 (414558,858036)	34.75 (23.56,49.67)	716192 (470439,1084538)	29.14 (19.19,44.32)	16.9% (1.9%,33.3%)	-0.414 (-0.816 - -0.011)
Low-middle SDI	396382 (272316,548633)	34.35 (23.55,48)	517206 (349525,756115)	27.64 (18.65,40.71)	30.5% (14.9%,43.6%)	-0.865 (-1.064 - -0.665)
Low SDI	209762 (139187,326279)	39.19 (26.45,59.82)	341930 (232579,498750)	30.13 (20.81,42.93)	63% (47.5%,79.5%)	-0.469 (-0.906 - -0.029)

AAPC: average annual percentage change; ASIR: age-standardized incidence rate; GBD: global Burden of Disease; SDI: sociodemographic index (*P < 0.05)

**Fig 1:**
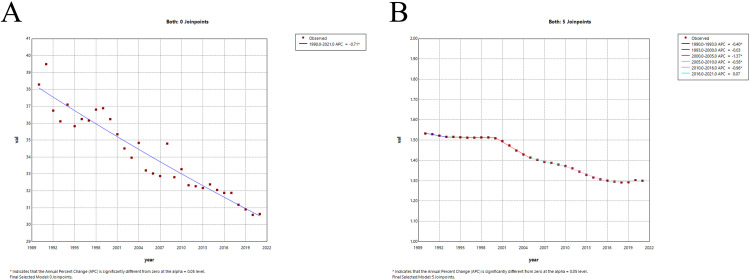
Joinpoint regression analysis of the global disease burden of hip dislocation from 1990 to 2021. **(A)** ASIR, **(B)** ASYR. ASIR: age-standardized incidence rate; ASYR: age-standardized YLD rate; YLDs: years lived with disability (*P < 0.05).

In 2021, the global ASIR of hip dislocation was 30.63 per 100,000 (95% UI, 20.65 to 44.8), with a corresponding ASYR of 1.3 per 100,000 (95% UI, 0.68 to 2.26). From 1990 to 2021, the global AAPC values for the ASIR and ASYR were −0.712 (95% confidence interval [CI], −0.79 to −0.634) and −0.535 (95% CI, −0.59 to −0.481), respectively (Fig 1).

### Regional trends

In 2021, Central Europe had the highest ASIR of hip dislocation (S2 Table in S1 File; 65.99 per 100,000 [95% UI, 41.13 to 102.11], followed by Eastern Europe (65.54 per 100,000 [95% UI, 42.63 to 98.45])), while Western Sub-Saharan Africa had the lowest ASIR (19.76 per 100,000 [95% UI, 13.85 to 27.11). Between 1990 and 2021, the ASIR for hip dislocation trended downwards in all global regions, except Oceania (AAPC, 0.026 [95% CI, −0.44 to 0.494]) and the Caribbean (AAPC, 0.311 [95% CI, −0.299 to 0.926]). During this period, the region with the most significant decrease in the ASIR was Eastern Sub-Saharan Africa (AAPC, −1.629 [95% CI, −2.292 to −0.961]).

In 2021, Eastern Europe had the highest ASYR for hip dislocation (S3 Table in S1 File) of 2.17 per 100,000 (95% UI, 1.11 to 3.75), followed by North Africa and the Middle East (1.98 per 100,000 [95% UI, 0.97 to 3.65]), and Central Europe (1.93 per 100,000 [95% UI, 0.99 to 3.36]). Western Sub-Saharan Africa (0.8 per 100,000 [95% UI, 0.41 to 1.41]) and Southern Sub-Saharan Africa (0.97 per 100,000 [95% UI, 0.51 to 1.72]) had the lowest ASYR values. Between 1990 and 2021, the Caribbean had the largest increase in the ASYR (AAPC, 1.209 [95% CI, 1.066 to 1.352]) while Southern Sub-Saharan Africa had the largest decrease in the ASYR (AAPC, −1.241 [95% CI, −1.314 to −1.167]).

### National trends

**Fig 2:**
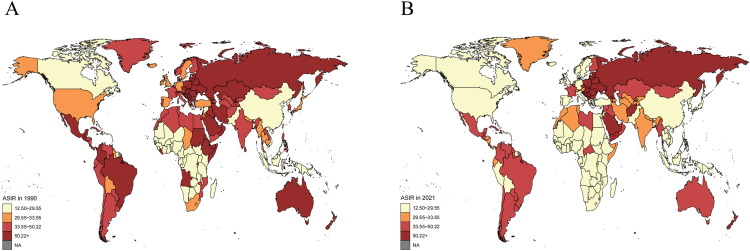
ASIR for hip dislocation per 100,000 per country in (A)1990 and (B)2021. ASIR: age-standardized incidence rate.

Considerable variation in the ASIR for hip dislocation was observed among countries in 2021 (S4 Table in S1 File; Fig 2). Afghanistan had the highest ASIR (157.41 per 100,000 [95% UI, 78.59 to 322.83]), followed by Yemen (93.92 per 100,000 [95% UI, 53.04 to 182.95]). The lowest ASIR values were observed in Kiribati (12.57 per 100,000 [95% UI, 8.63 to 17.94]), Tonga (14.56 per 100,000 [95% UI, 9.63 to 21.41]), and Fiji (15.7 per 100,000 [95% UI, 10.61 to 22.53]). In the period between 1990 and 2021, the ASIR for hip dislocation increased most significantly in Afghanistan and Yemen and decreased most substantially in Kuwait, Ethiopia, and Eritrea.

In 2021, the highest ASYRs for hip dislocation (S5 Table in S1 File; S1 Fig in S1 File) were observed in Afghanistan (9.03 per 100,000 [95% UI, 2.61 to 24.67]), Syria (5.85 per 100,000 [95% UI, 2.17 to 12.88]), and Eritrea (5.84 per 100,000 [95% UI, 1.87 to 14.76]). The lowest ASYRs for hip dislocation were reported in Madagascar (0.54 per 100,000 [95% UI, 0.28 to 0.92]), Malawi (0.56 per 100,000 [95% UI, 0.29 to 0.96]), and Tonga (0.56 per 100,000 [95% UI, 0.29 to 0.98]). Between 1990 and 2021, in the largest increases in ASYR occurred in Haiti, Syria, Rwanda, and Burundi, while the largest decreases in ASYR occurred in Korea, Lebanon, Estonia, and Latvia.

### Age-sex-specific patterns

**Fig 3:**
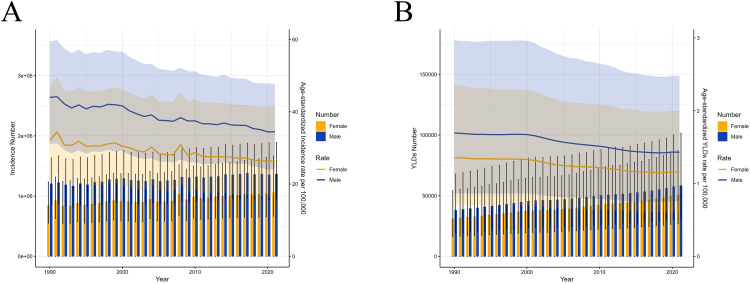
Incidence, YLDs, ASIR, and ASYR of hip dislocation among males and females from 1990 to 2021. (A) incident cases and ASIR; **(B)** YLDs and ASYR. Bar charts represent counts; lines represent age-standardized rates. ASIR: age-standardized incidence rate; ASYR: age-standardized YLD rate; YLDs: years lived with disability.

**Fig 4:**
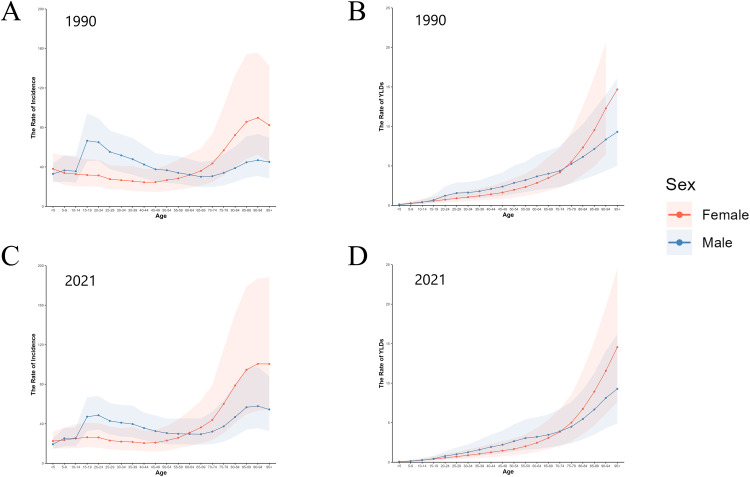
Incidence and YLDs for hip dislocation in different age groups of males and females in 1990 and 2021. **(A)** incidence in 1990; **(B)** YLDs in 1990; **(C)** incidence in 2021; **(D)** YLDs in 2021. YLDs: years lived with disability.

The global incidence and YLDs of hip dislocation for all age groups and among both males and females from 1990 to 2021 are presented in S6-S7 Tables in S1 File and summarized in Figs 3 and 4. The incidence and YLDs for hip dislocation were higher in males than in females across all age groups, with similar trends observed in both sexes (Fig 3). The ASRs also tended to be higher in males than in females. Between 1990 and 2021, the incidence of hip dislocation fluctuated in both sexes but showed an overall upward trend; however, the ASIRs of hip dislocation exhibited a downward trend during the same period.

The trends in the incidence of hip dislocation varied across age groups and between sexes (Fig 4; S2 Fig in S1 File). As shown in Fig 4, the highest incidence of hip dislocation in 1990 was observed among adolescent males, particularly those aged 15–19 years. In 2021, adolescence remained a period of elevated risk for males, but the peak incidence among males has shifted to the 85 + age group. Additionally, a second incidence peak emerged at 20–24 years. Conversely, the incidence of hip dislocation among females increased with age. Furthermore, the YLDs of hip dislocation increased with age among both males and females, and was particularly high for older age groups of both sexes.

### Injury patterns

The GBD 2021 identified various causes of hip dislocation; the ten most prevalent causes are listed in S8 Table in S1 File. Falls were consistently identified as the leading cause in terms of both incidence and YLDs. The ASIR for hip dislocation due to falls in the total global population was 15.09 (95% UI, 7.2 to 27.92) in 1990 and 14.77 (95% UI, 7.14 to 27.67) in 2021 and the corresponding ASYR was 0.6 (95% UI, 0.31 to 1.05) in 1990 and 0.58 (95% UI, 0.3 to 1) in 2021. The all-cause ASRs exhibited a slight downward trend between 1990 and 2021.

**Fig 5:**
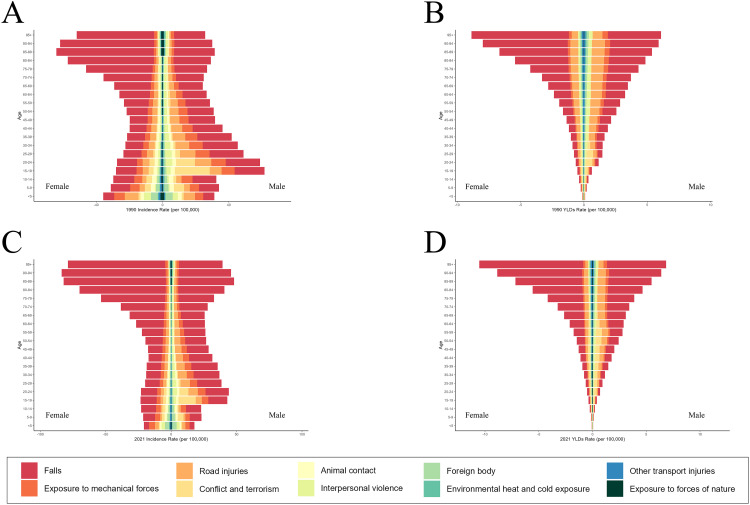
The 10 leading causes associated with the incidence and YLDs of hip dislocation for different age groups in 1990 and 2021. **(A)** incidence in 1990; **(B)** YLDs in 1990; **(C)** incidence in 2021; **(D)** YLDs in 2021. YLDs: years lived with disability.

Between 1990 and 2021, the global incidence of hip dislocation from non-fall causes (Fig 5A and 5C) decreased across all age groups, with more pronounced reductions in the younger age groups. However, the incidence of hip dislocations due to falls increased in most age groups of females, especially among elderly females. In contrast, the incidence of hip dislocations due to falls decreased in males under 45 but increased in males over 45. Moreover, the incidence of hip dislocations due to police conflict and executions increased in adolescents and young adults of both sexes between 1990 and 2021. In addition, the global YLDs associated with hip dislocations tended to decrease among the 65 + group between 1990 and 2021 (Fig 5B and 5D).

### Relationships between the ASRs and SDI

**Fig 6:**
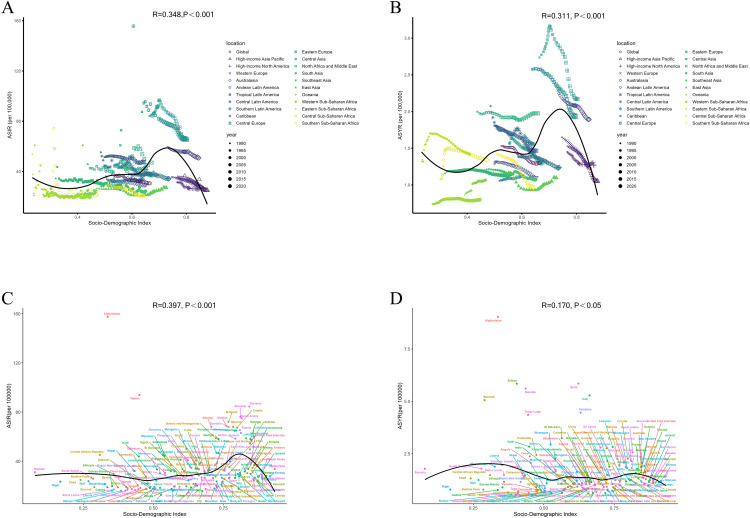
ASIR (A) and ASYR (B) of hip dislocation for 21 GBD regions by SDI from 1990 to 2021. **ASIR (C) and ASYR (D) of hip dislocation for 204 countries and territories by SDI in 2021.** The black line shows the trendline fit. The R- and p-values were calculated by Pearson’s correlation analysis. ASIR: age-standardized incidence rate; ASYR: age-standardized YLD rate; GBD, Global Burden of Disease Study; SDI, sociodemographic index; YLDs: years lived with disability.

At both regional and national levels, statistically significant non-linear relationships were observed between the ASRs of hip dislocation and SDI (Fig 6). S-shaped trends in the ASRs of hip dislocation were evident across the 21 GBD regions, as the SDI generally increased from 1990 to 2021 (Fig 6A and 6B). The higher ASRs observed in Central and Eastern Europe for the period 1990–2021 created an inflection point in the fitted curve, which subsequently declined with increasing SDI. This trend became less pronounced at the country level in 2021 (Fig 6C and 6D). It is noteworthy that several countries and regions, including Afghanistan and Yemen, reported ASIRs that exceeded the values expected based on their SDI.

### Prediction of the global burden of hip dislocation

**Fig 7:**
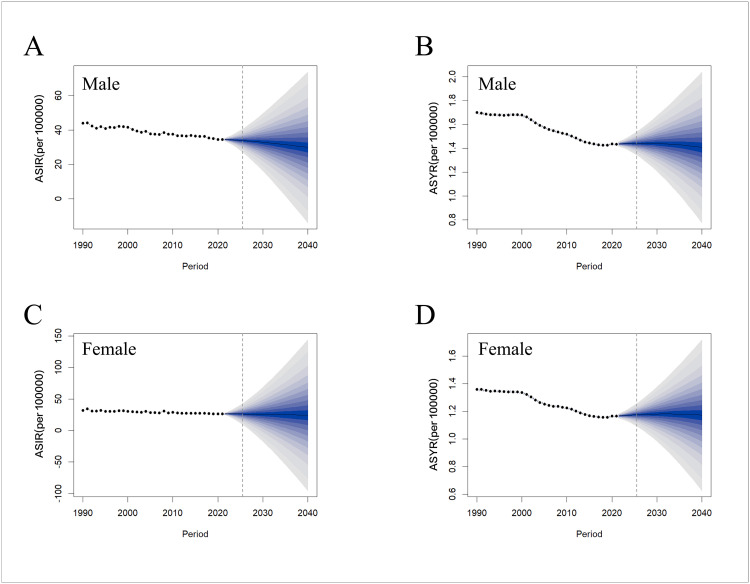
Observed and BAPC model-predicted global trends in the ASIR and ASYR for hip dislocation by sex from 1990 to 2040. **(A)** ASIR and **(B)** ASYR for males. **(C)** ASIR and **(D)** ASYR for females. ASIR: age-standardized incidence rate; ASYR: age-standardized YLD rate; BAPC: Bayesian Age-Period-Cohort; YLDs: years lived with disability.

To predict the future trends in the disease burden of hip dislocation from 2021 onwards, a Bayesian age-period-cohort model was employed to predict the global ASRs for both sexes from 2021 to 2040 (Fig 7). The model predicted a consistent decline in the ASIRs for both sexes, with the ASIR for males predicted to decrease from 34.51 per 100,000 in 2021 to 29.89 per 100,000 in 2040 and the ASIR for females predicted to decrease from 26.48 per 100,000 in 2021 to 24.3 per 100,000 in 2040. The ASYR for males is predicted to decline from 1.43 per 100,000 to 1.41 per 100,000 between 2021 and 2040, whereas the ASYR for females is predicted to remain at 1.17 per 100,000.

## Discussion

Between 1990 and 2021, there was a notable increase in the global incidence and YLDs of hip dislocation. However, the contrasting declines in the ASIR and ASYR for this injury over the same period can be attributed to a number of factors, including the increase in population, trends in population ageing, advances in medical technology, and improvements in public health measures. Over the past three decades, the incidence of hip dislocation has increased by more than 18%, primarily due to increased longevity, population ageing, and changes in lifestyle [20]. However, the rise in the number of new cases is the primary driver of the increase in the associated YLDs, which indicates a strong need for continued vigilance to prevent this severe injury.

Our data also indicate that variations in sociodemographic development may significantly contribute to the epidemiological imbalance of hip dislocation observed globally. Globally, the ASRs for hip dislocation and the associated YLDs exhibited a consistent pattern of initial increases followed by declines from 1990 to 2021. During this period, the incidence of hip dislocation tended to decrease in the majority of regions included in this study; this trend was more pronounced in regions with higher SDI values, especially in Central and Eastern Europe. This finding may be attributed to the fact that regions with higher SDI values typically place heightened emphasis on public health and safety, which in turn is reflected in the implementation of more comprehensive preventive measures and systems. For instance, these districts proactively encourage or legally enforce the utilization of personal protective equipment, including seat belts and safety nets in high-risk activities, with the objective of reducing the prevalence of traffic accidents and falls [21]. Furthermore, these regions are usually equipped with more sophisticated medical technology and robust emergency response systems [22] and offer enhanced rehabilitation services, which would result in more precise diagnosis and effective treatment, a lower risk of complications and sequelae, and thus reduced YLDs for patients who suffer hip dislocation.

Despite the observed decline in the ASRs of hip dislocation in high SDI regions between 1990 and 2021, the ASRs in these regions remain relatively high. In particular, the ASRs for Central and Eastern Europe were significantly higher than expected based on their SDI values. Firstly, individuals residing in regions with a high SDI are more inclined to seek specialized medical assistance following a hip dislocation, which would lead to high rates of reporting. In addition, these countries frequently possess well-developed medical and rehabilitation systems [3], which not only guarantee accurate diagnosis and documentation in a timely manner, but also facilitate the rehabilitation process. Furthermore, countries with higher SDI typically exhibit more pronounced demographic trends of ageing populations [1], characterized by higher proportions of older individuals who are more susceptible to hip dislocation. In contrast, in areas with low SDI, a significant proportion of the population may be unable to access healthcare services due to a lack of available resources and financial constraints. This may result in incomplete data on the number of cases of hip dislocation, which in turn would affect the accuracy and completeness of the data. Moreover, in countries with higher SDI values, the prevalence of industrial facilities and mechanical equipment [23] have resulted in a significant increase in the incidence of hip dislocation due to fall accidents and exposure to mechanical forces. Collectively, these factors may contribute to the elevated ASRs in these high-SDI regions. Moreover, increases in the ASIRs of hip dislocation were also observed in some developing regions with low and medium SDI values, such as Oceania and the Caribbean. This may be related to accelerated urbanization, increased risk of traffic accidents, and an increase in hospital visits due to improvements in healthcare. Furthermore, the lower economic status of these regions may result in a higher prevalence of high-risk occupations, and inadequate health and safety education and regulations may contribute to a lack of awareness or enforcement of preventive safety measures, and thereby increase the probability of injury. However, these regions continue to experience deficiencies in their healthcare resources and uneven levels of care, as well as socioeconomic disparities that may impact the accessibility of health services. This variability underscores the significance of optimal healthcare resource allocation and development of public health policies to reduce the incidence of hip dislocation and improve patient outcomes, especially in regions with low and medium SDI values.

Furthermore, across 204 countries and regions, the ASIR and ASYR of hip dislocation exhibited a significant, non-linear correlation with the SDI, although this relationship was characterized by complex regional trends. For example, the ASRs for Yemen and Afghanistan were significantly higher than expected based on the overall trend. This may be attributed to three factors. Firstly, there are high proportions of rural populations in Yemen (61.5%) and Afghanistan (70.8%) according to a World Bank report in 2021 [24]. Falls are more common among the elderly in rural areas than in non-rural areas [25,26], and falls are a significant cause of hip dislocation in the elderly [15]. Secondly, the World Bank reported that per capita health expenditure in 2021 was $70 in Afghanistan, while Yemen was heavily reliant on external funding. Both of these figures are considerably below the global average. Finally, between 1990 and 2021, both Yemen and Afghanistan were unfortunately affected by periods of armed conflict [27,28]. The occurrence of ongoing conflicts and violent incidents has been linked to an elevated risk of hip dislocation, particularly among younger individuals. Thus, the combined scourge of war and the absence of adequate healthcare makes it very challenging to mitigate the burden of hip dislocation in Afghanistan and Yemen. In contrast, Kiribati, Tonga, and Fiji have lower than average rates of hip dislocation, likely due to their low levels of traffic, flat terrain, and lifestyles emphasising light work and socializing over risky activities such high-risk sports. In addition, the noteworthy increase in the incidence of hip dislocation in the United Arab Emirates can be primarily attributed to the substantial population growth that occurred between 1990 and 2021; during this period, the nation’s population expanded from approximately 1.9 million to 9.365 million.

Globally, the incidence of hip dislocations and associated YLDs have remained consistently higher among males than females over the last thirty years. The incidence of hip dislocations in male’s peaks in early adulthood, then declines, and increases again in older age. Male predominance in hip dislocation is evident in two large-scale national datasets. Weber et al. [29] found that 79% of 1359 traumatic hip dislocations recorded in Germany (2002–2019) through the TraumaRegister DGU® occurred in males, while Moran et al. [11] reported that 81% of 102 sport-related hip dislocations in the US (2010–2019) from the U.S. National Electronic Injury Surveillance System (NEISS) database were male patients. These findings consistently align with our study’s results, demonstrating higher male susceptibility to hip dislocation. This may be attributed to the fact that men are more prone to occupying positions that entail elevated risk. The elevated rate of hip dislocations in young males is likely attributed to their increased participation in hazardous jobs and activities, including heavy lifting, operating machinery, and engaging in extreme sports, all of which are associated with a higher risk of sustaining hip joint injuries or dislocations. However, this trend is not consistent across all age groups. While national databases highlight male predominance in trauma overall, our analysis reveals a shift in elderly populations: elderly females exhibit higher incidence than elderly males. This discrepancy may stem from age-specific risk factors, including osteoporosis and fall-related mechanisms. The incidence of osteoporosis significantly increases in elderly females, particularly postmenopausal females, due to a dramatic decrease in estrogen levels. The resulting reduction in bone density is especially pronounced in the hip area, which heightens the risk of fractures and dislocations. Falls represent a significant risk factor for hip dislocation in the elderly population, and elderly females are particularly vulnerable due to weaker muscle strength, balance, and coordination [30].

Thus, while hip dislocation was predominantly assumed to occur among young people [11], our analysis indicates a noticeable shift toward an older demographic between 1990 and 2021. The elderly, particularly females, constitute a significant risk group, warranting targeted preventive strategies. In the context of aging populations, hip dislocation may emerge as an increasingly severe public health concern. Another high-risk group requiring attention is individuals who are overweight or obese. Research indicates that excess body weight elevates fall risk [31] and increases the risk of post-operative dislocation after hip replacement [8]. Given the global rise in obesity rates [32], these findings underscore the need for tailored fall prevention programs for overweight individuals, particularly when combined with age-related risk factors.

Our research indicates that falls are the leading cause of hip dislocation worldwide, followed by traffic accidents and exposure to mechanical forces. It is particularly concerning that traumatic hip dislocation caused by falling is disproportionately more common among the elderly population, especially among females. As the global population ages, there is an urgent need to implement additional preventive measures to reduce the incidence of falls among the elderly and mitigate the potential consequences of these injuries [32]. The prevention of falls among the elderly necessitates a unified approach involving individuals, families, communities, and the healthcare system and involves implementation of measures to enhance home safety, performance of fall risk assessments, and health education [33]. Furthermore, hip dislocation resulting from motor vehicle accidents frequently results in more extensive damage [34]. Therefore, a series of proactive measures need to be implemented to reduce the incidence of hip dislocation globally, such as enhancing driver education and training, raising awareness of safe driving practices, developing and implementing emergency response plans, and revising traffic safety laws and regulations.

Our BAPC forecasting model predicted that the global ASIR of hip dislocation will decline after 2021, likely due to increased emphasis on healthy living practices, such as regular exercise and avoidance of risky activities, coupled with heightened health awareness and advancements in medical technology. Notably, the ASYR of hip dislocation is declining in males but is projected to remain stable in females, indicating that the global burden of hip dislocation among females warrants our attention. This trend may reflect increase in the aging female population [8] and disparities in healthcare access [35]. Consequently, there is a need to prioritize preventive measures for elderly females. Policymakers could address these trends by enhancing healthcare accessibility and ensuring reasonable distribution of medical resources across genders and age groups, with a special focus on community health centers. They should also encourage community, family, and volunteer involvement to provide social support and emotional care for elderly females. Strengthening public health policies that target hip health, including health promotion, fall prevention, and early intervention, is also crucial. Additionally, modern technology, such as wearable devices to monitor the activity of the elderly and prevent falls and hip injuries, may help to further alleviate the burden of hip dislocation on individuals and society. Based on our findings, several areas warrant further investigation to inform future work in this domain, including establishing clinical registries for injury surveillance at the national level, conducting more granular analyses by injury type or cause, exploring preventative measures such as traffic safety initiatives and fall-prevention programs, and examining the public health implications of demographic changes on injury burden and cost modeling. Addressing these areas will contribute to a more comprehensive understanding of the epidemiology of hip dislocation and inform effective public health interventions.

## Limitations

This study has several limitations. Firstly, the GBD database is primarily comprised of data extracted from national and regional reports and publications, rather than official national reports. Thus, there may be issues related to the completeness, accessibility, and quality of the data in the GBD database. Secondly, the GBD data does not distinguish between different types of hip dislocation, thus we could not conduct an in-depth analysis of different subtypes of hip dislocation. In addition, the accuracy of our predictions for hip dislocation over the next 20 years depends on a range of factors, many of which are currently unknown. Consequently, the precision of the forecasts may be affected by uncertainties.

## Conclusion

The global burden of hip dislocation has increased over the past three decades, with relatively larger disease burdens in some high SDI regions and low-income countries. Moreover, the disease burden of hip dislocation is expected to increase in the future, particularly among elderly females, due to the trends in population aging. Therefore, appropriate preventive measures need to be developed to mitigate the incidence and burden of hip dislocation.

## Supporting information

S1 File All supplementary figures and tables (S1–S2 Figs, S1–S8 Tables) are provided in S1 File. (DOCX)

## Abbreviations

APC: Annual percentage change
AAPC: Average annual percentage change
ASR: Age-standardized rate
ASIR: Age-standardized incidence rate
ASYR: Age-standardized YLD rate
BAPC: Bayesian Age-Period-Cohort
CI: Confidence interval
GBD: Global Burden of Disease
SDI: Sociodemographic index
UI: Uncertainty intervals
YLD: Years lived with disability
