# Supplementary material for: Global, regional, and national burden of hip dislocation, 1990–2021, and Bayesian age-period-cohort predictions: Systematic analysis of the Global Burden of Disease study, 2021
Source: PLoS One. 2026 Jan 15;21(1):e0340294. doi: 10.1371/journal.pone.0340294 (PMC12806847; doi:10.1371/journal.pone.0340294)
Supplement: S1 File — (DOCX) [file pone.0340294.s001.docx]

***Global, regional, and national burden of hip dislocation, 1990-2021, and Bayesian age-period-cohort predictions: systematic analysis of the Global Burden of Disease study, 2021.***

**Contents**

**S1 Fig.……………………………………………………………………………………….2**

**S2 Fig………………………………………………………………………………………..3**

**S1 Table……………………………………………………………………………………. 4**

**S2 Table……………………………………………………………………………………. 5**

**S3 Table……………………………………………………………………………………. 6**

**S4 Table……………………………………………………………………………………. 7**

**S5 Table……………………………………………………………………………………. 14**

**S6 Table……………………………………………………………………………………. 21**

**S7 Table……………………………………………………………………………………. 22**

**S8 Table……………………………………………………………………………………. 23**


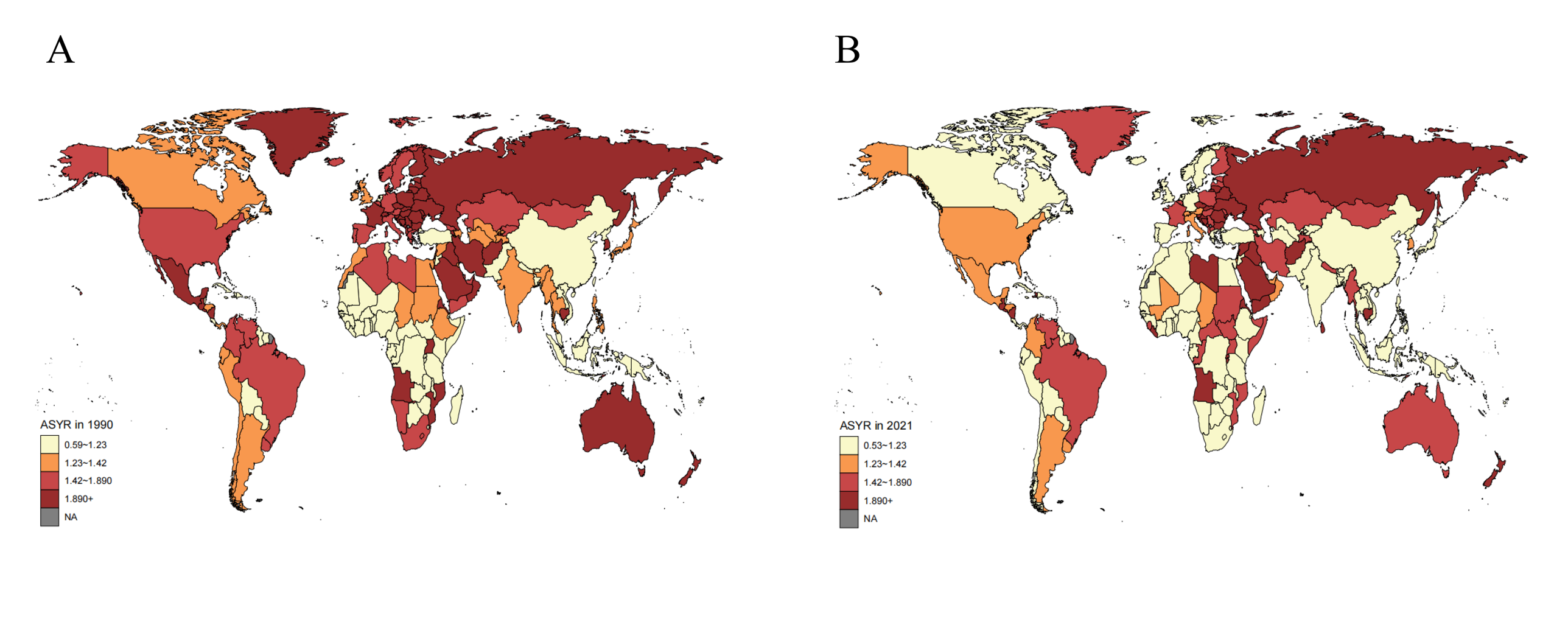


**S1 Fig. ASYR for hip dislocation per 100,000 per country in (A)1990 and (B)2021.** ASYR: age-standardized incidence rate


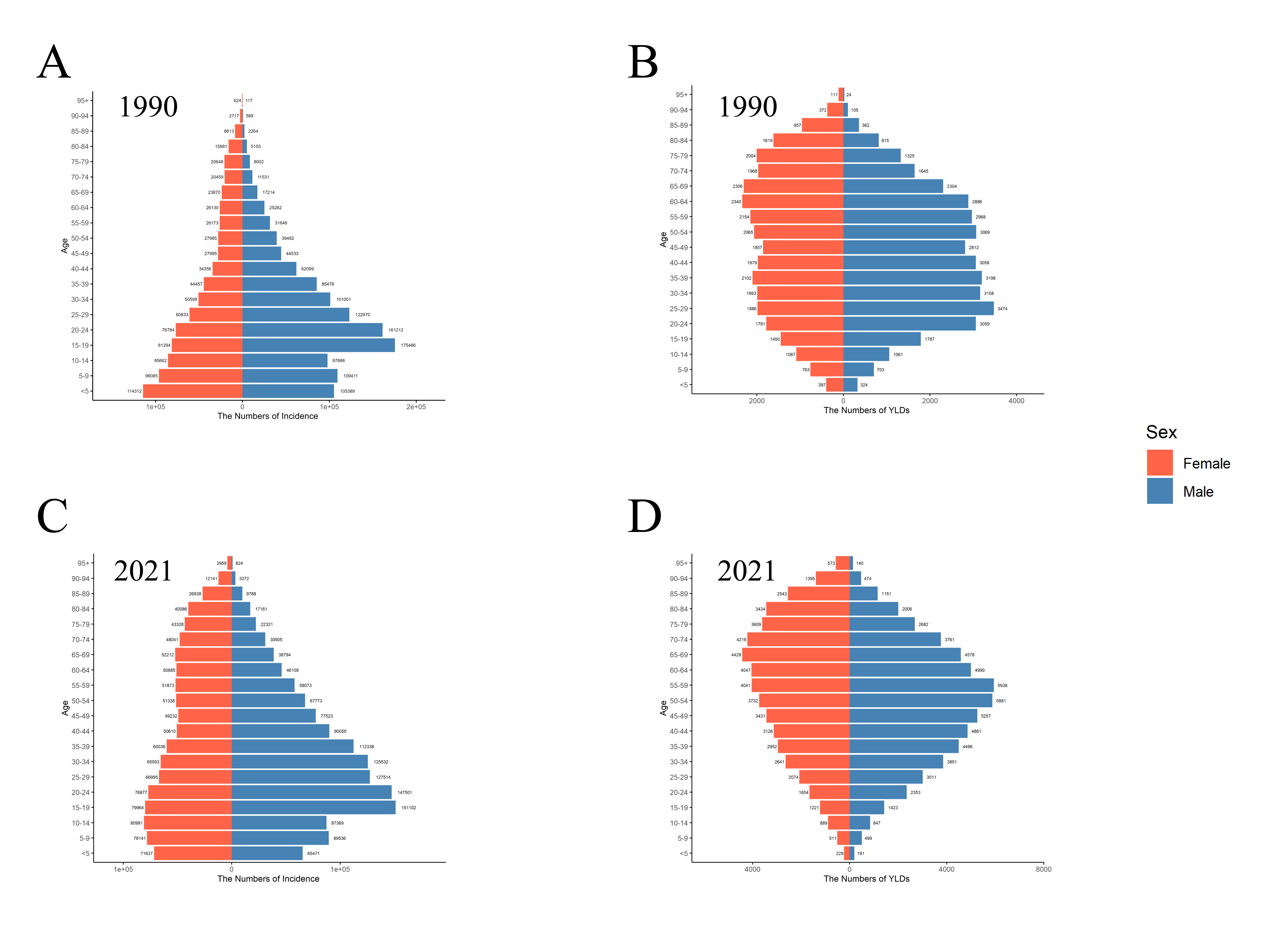


**S2 Fig. Incidence and YLDs number for hip dislocation in different age groups of males and females in 1990 and 2021.** (A) incidence in 1990; (B) YLDs in 1990; (C) incidence in 2021; (D) YLDs in 2021. YLDs: years lived with disability

**S1 Table. YLDs and ASYR of hip dislocation in 1990 and 2021, and the AAPC values for 1990 to 2021, stratified by global and SDI regions**

| **Characteristic** | **1990** | | **2021** | | **1990–2021** | |  |
| --- | --- | --- | --- | --- | --- | --- | --- |
|  | **YLDs** | **ASYR per 100,000** | **YLDs** | **ASYR per 100,000** | **Change of number** | **AAPC** |  |
|  |  |  |  |  |  |  |  |
|  | **n (95% UI)** | **n (95% UI)** | **n (95% UI)** | **n (95% UI)** | **n (95% UI)** | **n (95% CI)** |  |
| Global | 69430 (35716,120858) | 1.53 (0.8,2.68) | 109146 (57181,190161) | 1.3 (0.68,2.26) | 57.2% (50.9%,66.4%) | -0.535 (-0.590,-0.481)* |  |
| SDI regions |  |  |  |  |  |  |  |
| High SDI | 16677 (8677,28446) | 1.63 (0.84,2.79) | 22281 (11860,37471) | 1.3 (0.69,2.21) | 33.6% (27.9%,38.3%) | -0.717 (-0.767,-0.668)* |  |
| High-middle SDI | 18560 (9684,31875) | 1.79 (0.93,3.07) | 23431 (12293,40320) | 1.34 (0.7,2.3) | 26.2% (21.8%,31.5%) | -0.889 (-1.010,-0.767)* |  |
| Middle SDI | 18810 (9760,33099) | 1.35 (0.7,2.35) | 33066 (17667,57288) | 1.24 (0.66,2.14) | 75.8% (67.7%,85.5%) | -0.264 (-0.328,-0.199)* |  |
| Low-middle SDI | 10417 (5461,18517) | 1.23 (0.64,2.16) | 18643 (9886,32928) | 1.14 (0.61,1.99) | 79% (67.5%,92.4%) | -0.270 (-0.337,-0.203)* |  |
| Low SDI | 4878 (2284,9869) | 1.28 (0.64,2.45) | 11611 (5444,22418) | 1.47 (0.7,2.77) | 138% (104.4%,181.7%) | 0.427 (0.274,0.580)* |  |

AAPC: average annual percent change; ASYR: age-standardized YLD rate; CI: Confidence interval; GBD: global Burden of Disease; SDI: sociodemographic index; UI: Uncertainty intervals (**P* < 0.05)

**S2 Table. Incident cases and ASIR of hip dislocation in 1990 and 2021, and the AAPC values for 1990 to 2021, stratified by GBD study regions**

| **Characteristic** | **1990** | | **2021** | | **1990–2021** | |  |
| --- | --- | --- | --- | --- | --- | --- | --- |
|  | **YLDs** | **ASYR per 100,000** | **YLDs** | **ASYR per 100,000** | **Change of number** | **AAPC** |  |
|  |  |  |  |  |  |  |  |
|  | **n (95% UI)** | **n (95% UI)** | **n (95% UI)** | **n (95% UI)** | **n (95% UI)** | **n (95% CI)** |  |
| GBD regions |  |  |  |  |  |  |  |
| Andean Latin America | 401 (203,717) | 1.28 (0.67,2.26) | 705 (360,1245) | 1.1 (0.56,1.94) | 75.8% (55.1%,98.2%) | -0.503 (-0.556,-0.450)* |  |
| Australasia | 508 (265,870) | 2.28 (1.19,3.92) | 839 (442,1457) | 1.89 (1,3.31) | 65% (53.6%,78.6%) | -0.597 (-0.632,-0.562)* |  |
| Caribbean | 317 (168,546) | 1.06 (0.56,1.81) | 787 (384,1554) | 1.56 (0.76,3.11) | 148.7% (89.9%,262.4%) | 1.209 (1.066,1.352)* |  |
| Central Asia | 903 (473,1551) | 1.57 (0.83,2.68) | 1214 (637,2069) | 1.3 (0.69,2.21) | 34.5% (21.4%,55.4%) | -0.577 (-0.720,-0.434)* |  |
| Central Europe | 3562 (1857,6172) | 2.57 (1.34,4.45) | 3299 (1721,5725) | 1.93 (0.99,3.36) | -7.4% (-11.6%,-2.1%) | -0.924 (-1.061,-0.786)* |  |
| Central Latin America | 2678 (1370,4716) | 2.07 (1.07,3.67) | 3702 (1952,6506) | 1.42 (0.75,2.48) | 38.3% (26.7%,49.4%) | -1.191 (-1.303,-1.079)* |  |
| Central Sub-Saharan Africa | 444 (216,840) | 1.08 (0.56,1.97) | 1251 (575,2452) | 1.34 (0.63,2.56) | 181.8% (136.9%,238.2%) | 0.708 (0.479,0.938)* |  |
| East Asia | 12463 (6577,21419) | 1.19 (0.63,2.04) | 21561 (11370,37324) | 1.1 (0.58,1.91) | 73% (63.6%,83.1%) | -0.266 (-0.475,-0.056)* |  |
| Eastern Europe | 7076 (3679,12368) | 2.74 (1.42,4.78) | 6213 (3227,10794) | 2.17 (1.11,3.75) | -12.2% (-15.6%,-7.9%) | -0.733 (-0.849,-0.617)* |  |
| Eastern Sub-Saharan Africa | 2026 (908,4092) | 1.32 (0.63,2.51) | 3976 (1765,8299) | 1.42 (0.63,2.93) | 96.2% (79.4%,110.4%) | 0.202 (-0.078,0.483) |  |
| High-income Asia Pacific | 2990 (1541,5265) | 1.53 (0.79,2.69) | 3581 (1854,6085) | 1.05 (0.55,1.82) | 19.8% (12.8%,26.4%) | -1.200 (-1.254,-1.147)* |  |
| High-income North America | 4738 (2433,8167) | 1.45 (0.75,2.51) | 7182 (3834,12249) | 1.27 (0.67,2.17) | 51.6% (40.5%,61.9%) | -0.439 (-0.495,-0.383)* |  |
| North Africa and Middle East | 5561 (2649,10788) | 2 (1,3.76) | 11620 (5681,21697) | 1.98 (0.97,3.65) | 108.9% (82%,137.5%) | -0.032 (-0.160,0.096) |  |
| Oceania | 37 (19,63) | 0.86 (0.45,1.49) | 110 (58,198) | 1.07 (0.56,1.9) | 196.4% (158.2%,248.5%) | 0.650 (0.530,0.769)* |  |
| South Asia | 8657 (4529,14944) | 1.16 (0.61,1.98) | 18382 (9607,31587) | 1.14 (0.6,1.97) | 112.3% (98.7%,134.1%) | -0.056 (-0.105,-0.008)* |  |
| Southeast Asia | 4488 (2259,8620) | 1.2 (0.61,2.17) | 7541 (3935,13838) | 1.07 (0.56,1.94) | 68% (44.7%,92.6%) | -0.397 (-0.531,-0.263)* |  |
| Southern Latin America | 653 (340,1115) | 1.38 (0.72,2.36) | 982 (510,1681) | 1.24 (0.64,2.11) | 50.3% (40%,62.3%) | -0.350 (-0.393,-0.307)* |  |
| Southern Sub-Saharan Africa | 548 (286,965) | 1.43 (0.76,2.48) | 696 (370,1247) | 0.97 (0.51,1.72) | 27.2% (19.6%,34.6%) | -1.241 (-1.314,-1.167)* |  |
| Tropical Latin America | 2165 (1140,3779) | 1.81 (0.95,3.14) | 3705 (2004,6363) | 1.47 (0.8,2.51) | 71.1% (59.2%,90.2%) | -0.678 (-0.714,-0.641)* |  |
| Western Europe | 8251 (4302,13901) | 1.65 (0.86,2.82) | 9209 (4866,15422) | 1.25 (0.65,2.13) | 11.6% (6.7%,16.1%) | -0.882 (-0.920,-0.845)* |  |
| Western Sub-Saharan Africa | 965 (498,1662) | 0.74 (0.38,1.27) | 2590 (1359,4636) | 0.8 (0.41,1.41) | 168.5% (137.9%,220.8%) | 0.255 (0.172,0.337)* |  |

AAPC: average annual percent change; ASYR: age-standardized YLD rate; CI: Confidence interval; GBD: global Burden of Disease; UI: Uncertainty intervals (**P* < 0.05)

**S3 Table. YLDs and ASYR of hip dislocation in 1990 and 2021, and the AAPC values for 1990 to 2021, stratified by GBD regions**

| **Characteristic** | **1990** | | **2021** | | **1990–2021** | |  |
| --- | --- | --- | --- | --- | --- | --- | --- |
|  | **YLDs** | **ASYR per 100,000** | **YLDs** | **ASYR per 100,000** | **Change of number** | **AAPC** |  |
|  |  |  |  |  |  |  |  |
|  | **n (95% UI)** | **n (95% UI)** | **n (95% UI)** | **n (95% UI)** | **n (95% UI)** | **n (95% CI)** |  |
| GBD regions |  |  |  |  |  |  |  |
| Andean Latin America | 401 (203,717) | 1.28 (0.67,2.26) | 705 (360,1245) | 1.1 (0.56,1.94) | 75.8% (55.1%,98.2%) | -0.503 (-0.556,-0.450)* |  |
| Australasia | 508 (265,870) | 2.28 (1.19,3.92) | 839 (442,1457) | 1.89 (1,3.31) | 65% (53.6%,78.6%) | -0.597 (-0.632,-0.562)* |  |
| Caribbean | 317 (168,546) | 1.06 (0.56,1.81) | 787 (384,1554) | 1.56 (0.76,3.11) | 148.7% (89.9%,262.4%) | 1.209 (1.066,1.352)* |  |
| Central Asia | 903 (473,1551) | 1.57 (0.83,2.68) | 1214 (637,2069) | 1.3 (0.69,2.21) | 34.5% (21.4%,55.4%) | -0.577 (-0.720,-0.434)* |  |
| Central Europe | 3562 (1857,6172) | 2.57 (1.34,4.45) | 3299 (1721,5725) | 1.93 (0.99,3.36) | -7.4% (-11.6%,-2.1%) | -0.924 (-1.061,-0.786)* |  |
| Central Latin America | 2678 (1370,4716) | 2.07 (1.07,3.67) | 3702 (1952,6506) | 1.42 (0.75,2.48) | 38.3% (26.7%,49.4%) | -1.191 (-1.303,-1.079)* |  |
| Central Sub-Saharan Africa | 444 (216,840) | 1.08 (0.56,1.97) | 1251 (575,2452) | 1.34 (0.63,2.56) | 181.8% (136.9%,238.2%) | 0.708 (0.479,0.938)* |  |
| East Asia | 12463 (6577,21419) | 1.19 (0.63,2.04) | 21561 (11370,37324) | 1.1 (0.58,1.91) | 73% (63.6%,83.1%) | -0.266 (-0.475,-0.056)* |  |
| Eastern Europe | 7076 (3679,12368) | 2.74 (1.42,4.78) | 6213 (3227,10794) | 2.17 (1.11,3.75) | -12.2% (-15.6%,-7.9%) | -0.733 (-0.849,-0.617)* |  |
| Eastern Sub-Saharan Africa | 2026 (908,4092) | 1.32 (0.63,2.51) | 3976 (1765,8299) | 1.42 (0.63,2.93) | 96.2% (79.4%,110.4%) | 0.202 (-0.078,0.483) |  |
| High-income Asia Pacific | 2990 (1541,5265) | 1.53 (0.79,2.69) | 3581 (1854,6085) | 1.05 (0.55,1.82) | 19.8% (12.8%,26.4%) | -1.200 (-1.254,-1.147)* |  |
| High-income North America | 4738 (2433,8167) | 1.45 (0.75,2.51) | 7182 (3834,12249) | 1.27 (0.67,2.17) | 51.6% (40.5%,61.9%) | -0.439 (-0.495,-0.383)* |  |
| North Africa and Middle East | 5561 (2649,10788) | 2 (1,3.76) | 11620 (5681,21697) | 1.98 (0.97,3.65) | 108.9% (82%,137.5%) | -0.032 (-0.160,0.096) |  |
| Oceania | 37 (19,63) | 0.86 (0.45,1.49) | 110 (58,198) | 1.07 (0.56,1.9) | 196.4% (158.2%,248.5%) | 0.650 (0.530,0.769)* |  |
| South Asia | 8657 (4529,14944) | 1.16 (0.61,1.98) | 18382 (9607,31587) | 1.14 (0.6,1.97) | 112.3% (98.7%,134.1%) | -0.056 (-0.105,-0.008)* |  |
| Southeast Asia | 4488 (2259,8620) | 1.2 (0.61,2.17) | 7541 (3935,13838) | 1.07 (0.56,1.94) | 68% (44.7%,92.6%) | -0.397 (-0.531,-0.263)* |  |
| Southern Latin America | 653 (340,1115) | 1.38 (0.72,2.36) | 982 (510,1681) | 1.24 (0.64,2.11) | 50.3% (40%,62.3%) | -0.350 (-0.393,-0.307)* |  |
| Southern Sub-Saharan Africa | 548 (286,965) | 1.43 (0.76,2.48) | 696 (370,1247) | 0.97 (0.51,1.72) | 27.2% (19.6%,34.6%) | -1.241 (-1.314,-1.167)* |  |
| Tropical Latin America | 2165 (1140,3779) | 1.81 (0.95,3.14) | 3705 (2004,6363) | 1.47 (0.8,2.51) | 71.1% (59.2%,90.2%) | -0.678 (-0.714,-0.641)* |  |
| Western Europe | 8251 (4302,13901) | 1.65 (0.86,2.82) | 9209 (4866,15422) | 1.25 (0.65,2.13) | 11.6% (6.7%,16.1%) | -0.882 (-0.920,-0.845)* |  |
| Western Sub-Saharan Africa | 965 (498,1662) | 0.74 (0.38,1.27) | 2590 (1359,4636) | 0.8 (0.41,1.41) | 168.5% (137.9%,220.8%) | 0.255 (0.172,0.337)* |  |

AAPC: average annual percent change; ASYR: age-standardized YLD rate; CI: Confidence interval; GBD: global Burden of Disease; UI: Uncertainty intervals (**P* < 0.05)

**S4 Table. Incident cases and ASIR of hip dislocation in 1990 and 2021, and the AAPC values for 1990 to 2021, for 204 countries and regions**

| **Countries or territories** | **1990** | | **2021** | | **1990–2021** | |  |
| --- | --- | --- | --- | --- | --- | --- | --- |
|  | **Incident cases** | **ASIR per 100,000** | **Incident cases** | **ASIR per 100,000** | **Change of number** | **AAPC** |  |
|  |  |  |  |  |  |  |  |
|  | **n (95% UI)** | **n (95% UI)** | **n (95% UI)** | **n (95% UI)** | **n (95% UI)** | **n (95% CI)** |  |
| Afghanistan | 5342 (3657,8429) | 50.22 (35.16,77.77) | 54894 (27509,113400) | 157.41 (78.59,322.83) | 927.7% (580%,1355.2%) | 3.829 (0.618,7.142)* |  |
| Albania | 2851 (1775,4533) | 81.29 (50.58,128.3) | 1661 (1054,2586) | 67.87 (42.66,106.48) | -41.7% (-48.1%,-32.7%) | -1.088 (-1.556,-0.617)* |  |
| Algeria | 10955 (7366,15775) | 40.75 (27.34,58.26) | 13076 (8626,19048) | 29.65 (19.51,43.14) | 19.4% (5.8%,35.3%) | -1.053 (-1.704,-0.397)* |  |
| American Samoa | 10 (7,15) | 21.49 (14.19,31.01) | 10 (6,14) | 20.2 (13.32,29.83) | -4.1% (-13.2%,6.3%) | -0.088 (-0.785,0.613) |  |
| Andorra | 19 (12,30) | 35.74 (22.38,54.69) | 34 (20,56) | 35.81 (22,57.12) | 76.9% (56.7%,99.2%) | 0.008 (-0.024,0.039) |  |
| Angola | 5862 (3520,10639) | 50.48 (31.09,90.05) | 5936 (4194,8254) | 18.25 (12.72,25.71) | 1.3% (-44.8%,57.9%) | -2.796 (-12.088,7.477) |  |
| Antigua and Barbuda | 19 (13,30) | 30.58 (20.16,47.64) | 28 (17,46) | 32.59 (20.44,52.16) | 46.8% (34.8%,59.8%) | 0.041 (-0.376,0.460) |  |
| Argentina | 12822 (8429,19028) | 38.59 (25.39,57.3) | 15034 (9844,22026) | 33.79 (22.07,50.3) | 17.2% (10.4%,23.6%) | -0.439 (-0.546,-0.331)* |  |
| Armenia | 1969 (1313,2900) | 56.05 (37.29,83.16) | 920 (629,1332) | 32.71 (22.35,47.85) | -53.3% (-56.4%,-49.6%) | -1.549 (-2.249,-0.844)* |  |
| Australia | 9688 (6184,14447) | 58.1 (37.34,85.8) | 12924 (8135,19248) | 49.57 (31.2,73.08) | 33.4% (22%,49.3%) | -0.509 (-0.547,-0.470)* |  |
| Austria | 3298 (2117,5062) | 41.09 (26.18,60.43) | 2974 (1828,4622) | 30.96 (19.37,46.71) | -9.8% (-19.1%,-2.6%) | -0.901 (-0.975,-0.826)* |  |
| Azerbaijan | 2991 (2037,4348) | 38.17 (26,55.91) | 3275 (2208,4819) | 31.85 (21.55,46.77) | 9.5% (1.8%,15.8%) | -1.009 (-1.808,-0.204)* |  |
| Bahamas | 66 (46,94) | 24.8 (17.35,35.53) | 96 (65,140) | 25.19 (17.17,36.59) | 44.4% (32.9%,57.6%) | 0.201 (-0.213,0.616) |  |
| Bahrain | 149 (103,211) | 27.52 (18.82,39.19) | 402 (266,589) | 25.57 (16.95,37.5) | 169% (142.2%,198.4%) | -0.203 (-0.504,0.098) |  |
| Bangladesh | 19951 (14220,27324) | 16.73 (11.7,23.26) | 26587 (17536,38852) | 15.81 (10.52,23.05) | 33.3% (13.1%,53.6%) | -0.188 (-0.814,0.441) |  |
| Barbados | 57 (40,85) | 22.56 (15.54,33.21) | 64 (43,96) | 23.13 (15.67,34.39) | 11.7% (2.1%,23.3%) | 0.125 (-0.136,0.386) |  |
| Belarus | 7361 (4718,11088) | 71.88 (45.71,108.45) | 6193 (3899,9605) | 67.94 (42.67,105.14) | -15.9% (-24.3%,-6.5%) | -0.178 (-0.290,-0.066)* |  |
| Belgium | 3931 (2566,5801) | 37.99 (24.96,55.46) | 4583 (2784,7277) | 34.44 (21.22,53.67) | 16.6% (2.3%,30.6%) | -0.318 (-0.467,-0.169)* |  |
| Belize | 68 (48,97) | 31.95 (22.53,45.77) | 144 (98,213) | 31.99 (21.58,46.99) | 111.4% (73.6%,143%) | -0.128 (-0.450,0.196) |  |
| Benin | 1027 (737,1422) | 19.5 (13.7,27.19) | 2572 (1824,3530) | 18.78 (13,26.44) | 150.5% (135.6%,167.3%) | -0.110 (-0.195,-0.025)* |  |
| Bermuda | 15 (10,21) | 25.28 (17.32,37.39) | 15 (10,23) | 25 (16.51,37.59) | 0.9% (-12.1%,17.6%) | -0.049 (-0.162,0.065) |  |
| Bhutan | 138 (93,199) | 21.91 (14.64,31.81) | 202 (131,304) | 27.61 (18.02,41.67) | 46.4% (24.9%,74.2%) | 0.416 (-0.055,0.889) |  |
| Bolivia (Plurinational State of) | 2179 (1553,3036) | 32.97 (23.3,46.08) | 3364 (2311,4861) | 28.5 (19.56,41.09) | 54.4% (39.4%,69.6%) | -0.541 (-0.593,-0.488)* |  |
| Bosnia and Herzegovina | 4085 (2611,6169) | 90.76 (57.94,137.38) | 1731 (1086,2714) | 58.58 (36.78,91.62) | -57.6% (-66.4%,-48.1%) | -0.987 (-4.742,2.916) |  |
| Botswana | 280 (197,385) | 20.64 (14.41,28.81) | 522 (365,732) | 21.43 (14.94,30.06) | 86.7% (69.1%,106%) | 0.174 (0.019,0.328)* |  |
| Brazil | 79145 (52257,118159) | 51.9 (34.26,77.27) | 90319 (59635,134377) | 40.47 (26.86,60.07) | 14.1% (7.7%,21.7%) | -0.850 (-1.090,-0.609)* |  |
| Brunei Darussalam | 94 (62,131) | 35.97 (23.8,49.39) | 130 (84,189) | 29.04 (18.87,41.86) | 38.7% (28.1%,48.7%) | -0.694 (-0.738,-0.650)* |  |
| Bulgaria | 7137 (4559,10804) | 87.56 (56.55,129.84) | 4259 (2601,6716) | 72.04 (44.94,112.38) | -40.3% (-46.3%,-34.3%) | -0.628 (-0.699,-0.558)* |  |
| Burkina Faso | 1996 (1408,2777) | 19.81 (13.72,27.94) | 6171 (4263,8756) | 25.73 (18.11,35.85) | 209.2% (152.3%,351.8%) | 1.136 (0.604,1.670)* |  |
| Burundi | 1163 (829,1636) | 20.12 (14.28,28.16) | 2565 (1850,3449) | 18.87 (13.49,25.88) | 120.6% (101.9%,154.9%) | -0.403 (-10.267,10.545) |  |
| Cabo Verde | 76 (55,108) | 20.35 (14.41,28.6) | 109 (75,158) | 19.87 (13.63,28.8) | 43.2% (26%,61.8%) | -0.100 (-0.186,-0.013)* |  |
| Cambodia | 3515 (2427,5138) | 33.17 (22.87,47.19) | 4393 (2830,6701) | 26.7 (17.27,41.39) | 25% (-16.3%,66.5%) | -0.414 (-1.861,1.054) |  |
| Cameroon | 1944 (1369,2665) | 18.2 (12.68,25.87) | 7013 (5011,9646) | 21.67 (15.27,30.01) | 260.7% (214.2%,356.8%) | 0.977 (0.512,1.445)* |  |
| Canada | 8400 (5558,11731) | 29.55 (19.49,41.31) | 10951 (7053,16446) | 24.28 (15.8,34.86) | 30.4% (12.2%,51.3%) | -0.629 (-0.675,-0.582)* |  |
| Central African Republic | 596 (428,835) | 20.89 (14.88,29.09) | 2807 (1835,4685) | 45.12 (30.04,71.91) | 371.1% (214.3%,729.3%) | 1.768 (0.946,2.597)* |  |
| Chad | 2085 (1411,3331) | 30.93 (20.88,48.66) | 4768 (3335,6733) | 24.7 (17.59,34.04) | 128.7% (94.9%,158.5%) | -0.479 (-1.165,0.212) |  |
| Chile | 4668 (3040,6755) | 34.45 (22.43,49.94) | 6328 (3931,9811) | 35.18 (21.99,53.52) | 35.6% (22.9%,47.8%) | 0.124 (-0.149,0.398) |  |
| China | 311602 (208484,452304) | 26.58 (17.65,38.88) | 410680 (254091,649132) | 27.56 (17.35,43.16) | 31.8% (14.2%,52.4%) | 0.037 (-0.875,0.957) |  |
| Colombia | 19189 (13065,27698) | 54.77 (37.21,78.21) | 15909 (10478,23148) | 33.81 (22.24,48.97) | -17.1% (-29.6%,-7.8%) | -1.583 (-2.240,-0.923)* |  |
| Comoros | 93 (66,129) | 19.39 (13.78,26.94) | 126 (88,178) | 17.24 (12.05,24.45) | 35.1% (25%,46%) | -0.079 (-0.545,0.389) |  |
| Congo | 479 (339,664) | 19.82 (13.79,27.77) | 892 (639,1224) | 17.06 (11.96,24.01) | 86.4% (74.7%,101.1%) | -1.167 (-2.197,-0.126)* |  |
| Cook Islands | 5 (3,7) | 24.92 (17.14,34.5) | 4 (2,5) | 19.94 (12.86,30.33) | -25.2% (-46.3%,-5.2%) | -0.251 (-0.832,0.334) |  |
| Costa Rica | 1205 (782,1840) | 38.15 (24.65,58.06) | 1573 (1023,2391) | 34.53 (22.3,53) | 30.5% (20.4%,40.6%) | -0.348 (-0.510,-0.186)* |  |
| Croatia | 4153 (2720,6184) | 86.53 (57.21,127.81) | 3235 (1922,5311) | 67.26 (41.44,106.65) | -22.1% (-39.2%,-3.2%) | -1.613 (-2.171,-1.052)* |  |
| Cuba | 3857 (2603,5684) | 35.03 (23.68,52.12) | 4796 (2994,7672) | 38.58 (25.09,59.32) | 24.3% (3.5%,50.9%) | 0.314 (0.252,0.376)* |  |
| Cyprus | 280 (184,397) | 36.56 (23.94,51.89) | 416 (265,628) | 30.22 (19.29,44.73) | 48.3% (32.4%,65.7%) | -0.613 (-0.736,-0.490)* |  |
| Czechia | 10704 (6797,16784) | 102.16 (65.73,158.13) | 7443 (4582,11946) | 70.58 (44.16,110.74) | -30.5% (-34.9%,-25.8%) | -1.199 (-1.379,-1.019)* |  |
| C么te d'Ivoire | 2398 (1713,3322) | 19.36 (13.61,26.92) | 5031 (3539,7077) | 18.54 (12.9,26.49) | 109.8% (96.7%,124.3%) | -0.147 (-0.527,0.236) |  |
| Democratic People's Republic of Korea | 3981 (2729,5742) | 19.04 (13.04,27.53) | 4440 (3085,6523) | 16 (11.14,23.05) | 11.5% (2%,22.1%) | -0.452 (-0.582,-0.322)* |  |
| Democratic Republic of the Congo | 8468 (5951,11712) | 20.41 (14.43,28.41) | 20709 (14879,28592) | 22.22 (15.99,29.77) | 144.6% (111.5%,209.1%) | 0.761 (-2.552,4.188) |  |
| Denmark | 2059 (1320,3013) | 35.9 (23.28,52.49) | 1618 (1010,2479) | 25.98 (16.42,39.02) | -21.4% (-28.3%,-16.4%) | -1.042 (-1.135,-0.949)* |  |
| Djibouti | 151 (99,248) | 32.18 (22.18,50.99) | 218 (154,301) | 18.01 (12.68,25.02) | 44.7% (-15.8%,105%) | -1.264 (-1.815,-0.709)* |  |
| Dominica | 17 (12,25) | 22.85 (16.11,32.52) | 15 (10,22) | 23.55 (16.01,34.25) | -11% (-17.1%,-4.3%) | 0.375 (-0.141,0.893) |  |
| Dominican Republic | 1837 (1299,2599) | 23.99 (17.06,33.98) | 3120 (2148,4504) | 27.96 (19.29,40.45) | 69.8% (57.1%,81.5%) | 0.457 (0.216,0.698)* |  |
| Ecuador | 3498 (2404,4986) | 33.89 (23.17,48.35) | 6138 (4095,9040) | 33.5 (22.36,49.28) | 75.4% (62.1%,89.4%) | -0.134 (-0.326,0.058) |  |
| Egypt | 21457 (14595,29969) | 36.17 (24.6,50.77) | 29630 (20697,41410) | 27.92 (19.39,39.09) | 38.1% (22%,56.2%) | -0.664 (-0.945,-0.382)* |  |
| El Salvador | 4639 (2949,7581) | 75.31 (48.84,122.28) | 2412 (1615,3505) | 36.96 (24.73,53.56) | -48% (-70.2%,-23.4%) | -2.665 (-3.948,-1.364)* |  |
| Equatorial Guinea | 93 (66,129) | 20.57 (14.75,28.7) | 235 (163,326) | 16.15 (11.15,23.18) | 153.9% (126.6%,187.2%) | -0.837 (-0.920,-0.754)* |  |
| Eritrea | 9688 (4349,21647) | 241.83 (110.11,540.04) | 1216 (861,1696) | 19.09 (13.43,26.76) | -87.4% (-94.9%,-70.8%) | -9.211 (-16.008,-1.864)* |  |
| Estonia | 1379 (894,2050) | 89.23 (57.54,132.81) | 675 (433,1056) | 54.42 (34.78,85.65) | -51.1% (-55.2%,-47.6%) | -1.605 (-1.751,-1.459)* |  |
| Eswatini | 177 (128,242) | 21.49 (15.39,29.7) | 355 (247,494) | 28.61 (20.12,38.91) | 100.2% (59.2%,185.6%) | 0.867 (0.634,1.100)* |  |
| Ethiopia | 68965 (35114,142181) | 119.84 (62.43,243.72) | 34813 (21779,62356) | 29.45 (19.18,49.34) | -49.5% (-67.4%,-21.8%) | -5.587 (-9.815,-1.162)* |  |
| Fiji | 129 (87,183) | 16.74 (11.2,23.8) | 143 (96,204) | 15.7 (10.61,22.53) | 10.6% (3.4%,19.1%) | -0.402 (-0.684,-0.120)* |  |
| Finland | 2513 (1630,3679) | 47.68 (31.03,69.84) | 2287 (1395,3597) | 36.75 (22.64,57.14) | -9% (-19.9%,1.1%) | -0.838 (-1.033,-0.643)* |  |
| France | 26371 (17272,38445) | 42.45 (27.83,61.64) | 26373 (16497,40459) | 33.75 (21.26,51.58) | 0% (-10.3%,9.7%) | -0.728 (-0.850,-0.605)* |  |
| Gabon | 207 (145,285) | 20.94 (14.64,29.31) | 326 (227,457) | 18.59 (13.01,26.53) | 57.6% (47.9%,67.8%) | -0.435 (-0.491,-0.380)* |  |
| Gambia | 176 (123,246) | 17 (11.81,24.11) | 390 (273,555) | 16.87 (11.59,24.65) | 121.2% (105.5%,139.9%) | -0.006 (-0.220,0.208) |  |
| Georgia | 3002 (1925,4524) | 55.26 (35.43,82.98) | 1935 (1234,2966) | 58 (36.98,88.8) | -35.5% (-39.1%,-32.2%) | -0.066 (-0.625,0.497) |  |
| Germany | 26835 (17543,39498) | 32.69 (21.62,47.91) | 26758 (16504,41588) | 27.94 (17.51,42.02) | -0.3% (-10.6%,8.4%) | -0.505 (-0.554,-0.455)* |  |
| Ghana | 2655 (1859,3691) | 17.38 (12.12,24.72) | 5778 (4001,8212) | 17.37 (11.93,24.98) | 117.7% (101.7%,134.8%) | -0.005 (-0.236,0.226) |  |
| Greece | 3623 (2395,5190) | 35.17 (23.45,50.92) | 2659 (1753,3793) | 26.29 (16.85,38.32) | -26.6% (-31.6%,-21.1%) | -0.899 (-1.065,-0.731)* |  |
| Greenland | 23 (15,32) | 41.99 (28.18,59.82) | 19 (12,28) | 31.67 (20.89,46.91) | -16.5% (-26.1%,-6%) | -0.977 (-1.231,-0.722)* |  |
| Grenada | 25 (17,37) | 27.97 (19.2,41.3) | 30 (19,46) | 29.7 (19.39,45.49) | 20.6% (9%,32.2%) | 0.126 (-0.144,0.396) |  |
| Guam | 26 (18,39) | 18.93 (12.5,27.95) | 28 (18,43) | 17.58 (11.52,26.65) | 5.6% (-6%,16.6%) | -0.291 (-0.580,-0.001)* |  |
| Guatemala | 5683 (3806,8477) | 59.98 (39.89,88.18) | 7172 (4626,10727) | 44.29 (28.78,66.55) | 26.2% (-17.9%,62.7%) | -0.839 (-1.557,-0.117)* |  |
| Guinea | 1286 (915,1764) | 19.32 (13.56,26.66) | 2590 (1830,3559) | 18.8 (13.11,26.13) | 101.4% (90.2%,113.5%) | -0.073 (-0.581,0.437) |  |
| Guinea-Bissau | 243 (171,336) | 22.91 (16.05,31.9) | 392 (275,545) | 19.65 (13.67,27.73) | 61.4% (52.6%,70.7%) | -0.688 (-1.242,-0.132)* |  |
| Guyana | 239 (170,334) | 29.75 (20.88,41.98) | 263 (180,373) | 34.06 (23.35,48.43) | 9.9% (1.4%,18.6%) | 0.317 (0.043,0.591)* |  |
| Haiti | 2142 (1506,3051) | 31.58 (22.13,44.71) | 5892 (3940,8676) | 43.78 (29.25,64.7) | 175.1% (120.3%,302%) | -0.089 (-1.093,0.926) |  |
| Honduras | 2066 (1366,3036) | 38.22 (25.49,56.01) | 3448 (2313,5064) | 33.25 (22.45,48.19) | 66.9% (55.1%,83%) | -0.621 (-1.102,-0.138)* |  |
| Hungary | 10098 (6302,16124) | 93 (58.93,143.04) | 6407 (3934,10402) | 64.77 (40.39,100.84) | -36.6% (-39.7%,-33.9%) | -1.162 (-1.286,-1.038)* |  |
| Iceland | 87 (56,125) | 33.55 (21.53,48.17) | 99 (62,152) | 27.64 (17.41,41.78) | 14.5% (4.7%,23.7%) | -0.678 (-1.011,-0.344)* |  |
| India | 312341 (208680,450285) | 38.83 (25.59,56.81) | 434893 (271390,676027) | 31.67 (19.67,50) | 39.2% (16.5%,58.7%) | -0.669 (-0.922,-0.416)* |  |
| Indonesia | 50959 (34264,73220) | 27.54 (18.41,39.62) | 54031 (35985,79088) | 20.03 (13.4,29.61) | 6% (-5%,18.6%) | -1.139 (-1.504,-0.773)* |  |
| Iran (Islamic Republic of) | 39095 (26547,55998) | 64.77 (44.59,91.69) | 27772 (18749,40113) | 32.68 (22.05,47.16) | -29% (-50.6%,-9%) | -1.491 (-1.772,-1.209)* |  |
| Iraq | 11747 (8513,16598) | 62.4 (45.84,87.86) | 18098 (12805,25567) | 42.87 (30.4,59.99) | 54.1% (33.1%,72%) | -1.444 (-6.827,4.250) |  |
| Ireland | 1119 (722,1617) | 31.04 (19.95,44.75) | 1344 (835,2052) | 27.27 (17.15,41.18) | 20.1% (7.4%,31.5%) | -0.423 (-0.486,-0.361)* |  |
| Israel | 1665 (1091,2330) | 33.11 (21.74,46.39) | 2942 (1909,4095) | 30.51 (19.92,42.92) | 76.7% (61.2%,96.8%) | -0.669 (-1.925,0.603) |  |
| Italy | 23303 (14813,35356) | 39.38 (25.37,58.81) | 17518 (10604,27675) | 27.47 (16.9,42.49) | -24.8% (-32.5%,-17.9%) | -1.163 (-1.314,-1.012)* |  |
| Jamaica | 746 (507,1121) | 29.76 (20.07,45.38) | 843 (575,1251) | 30.28 (20.7,44.81) | 13% (1.3%,29.2%) | 0.093 (-0.243,0.430) |  |
| Japan | 39671 (25493,56879) | 31.94 (20.62,45.21) | 31154 (20152,44198) | 23.47 (15.12,33.94) | -21.5% (-29%,-12.9%) | -1.162 (-1.345,-0.980)* |  |
| Jordan | 1191 (815,1681) | 29.65 (20.31,41.84) | 3019 (1995,4435) | 23.54 (15.51,34.76) | 153.4% (128.4%,184.2%) | -0.700 (-0.789,-0.612)* |  |
| Kazakhstan | 9364 (6179,13762) | 55.02 (36.23,81.24) | 8865 (5867,13405) | 46.86 (30.94,70.76) | -5.3% (-10.3%,0.4%) | -0.504 (-0.612,-0.395)* |  |
| Kenya | 4520 (3207,6323) | 19.47 (13.59,27.4) | 8557 (6073,12022) | 18.29 (12.68,25.72) | 89.3% (73.2%,105.2%) | -0.295 (-0.648,0.060) |  |
| Kiribati | 11 (8,16) | 14.74 (10.3,21.13) | 15 (11,22) | 12.57 (8.63,17.94) | 37.1% (23.5%,49%) | -0.989 (-1.474,-0.503)* |  |
| Kuwait | 2078 (1166,3888) | 115.2 (64.08,219.63) | 1435 (919,2198) | 29.6 (19.06,45.01) | -30.9% (-67%,25.7%) | -5.182 (-6.320,-4.031)* |  |
| Kyrgyzstan | 2248 (1501,3294) | 47.86 (32.26,70.71) | 2372 (1606,3397) | 33.52 (22.74,47.96) | 5.5% (-1.4%,15%) | -1.377 (-1.600,-1.153)* |  |
| Lao People's Democratic Republic | 1648 (1059,2841) | 37.31 (24.4,61.54) | 1409 (959,2005) | 19.14 (12.92,27.47) | -14.5% (-53.1%,22.8%) | -0.765 (-1.028,-0.501)* |  |
| Latvia | 2599 (1673,3889) | 98.25 (62.71,147.1) | 1056 (681,1644) | 58.6 (37.28,91.91) | -59.4% (-62.3%,-56.8%) | -1.702 (-1.860,-1.545)* |  |
| Lebanon | 2219 (1373,3845) | 71.7 (44.55,120.42) | 1477 (980,2120) | 26.38 (17.51,37.61) | -33.5% (-61.9%,2.7%) | -2.183 (-3.228,-1.126)* |  |
| Lesotho | 309 (223,424) | 19.58 (14.16,27.43) | 465 (338,653) | 24.15 (17.57,33.98) | 50.3% (34.4%,65.4%) | 0.691 (-0.406,1.800) |  |
| Liberia | 4161 (1958,9019) | 148.7 (71,322.22) | 858 (594,1207) | 16.08 (11.17,23.28) | -79.4% (-91.1%,-54.5%) | -2.921 (-18.067,15.024) |  |
| Libya | 1523 (1023,2209) | 34.72 (23.38,50.34) | 2517 (1737,3574) | 38.05 (26.48,54.91) | 65.2% (42.9%,99.5%) | 2.223 (-0.061,4.560) |  |
| Lithuania | 3236 (2067,4830) | 87.56 (55.73,130.17) | 1661 (1035,2577) | 60.28 (38.3,93.56) | -48.7% (-53.3%,-44.2%) | -1.219 (-1.357,-1.081)* |  |
| Luxembourg | 187 (121,263) | 46.24 (30.5,66.21) | 220 (138,335) | 32.18 (20.21,48.94) | 17.9% (2.2%,34.2%) | -1.163 (-1.263,-1.063)* |  |
| Madagascar | 2381 (1674,3356) | 18.39 (13.05,25.99) | 4717 (3396,6504) | 16.04 (11.27,22.35) | 98.1% (85.6%,118.6%) | -0.464 (-0.563,-0.365)* |  |
| Malawi | 1885 (1313,2643) | 17.55 (12.12,24.67) | 3094 (2147,4308) | 15.88 (10.84,22.74) | 64.1% (52.5%,78.3%) | -0.404 (-0.489,-0.319)* |  |
| Malaysia | 3713 (2546,5250) | 21.54 (14.66,30.6) | 6727 (4526,9790) | 20.9 (14.26,30.39) | 81.2% (62.9%,101.4%) | -0.189 (-0.241,-0.136)* |  |
| Maldives | 54 (36,79) | 24.88 (16.81,36.69) | 114 (74,174) | 21.65 (14.04,33.13) | 110.7% (72%,155.4%) | -0.417 (-0.914,0.081) |  |
| Mali | 2687 (1884,3869) | 28.44 (20.03,40.32) | 6064 (4301,8316) | 23.68 (16.81,32.6) | 125.7% (102.4%,146.8%) | -0.125 (-5.715,5.796) |  |
| Malta | 127 (81,186) | 34.51 (22.33,50.82) | 134 (84,204) | 29.6 (18.56,44.94) | 6.2% (-2.3%,14.8%) | -0.509 (-0.631,-0.386)* |  |
| Marshall Islands | 9 (6,12) | 20.05 (13.62,28.43) | 11 (7,15) | 19.24 (13.01,28.1) | 24.9% (15.4%,36%) | -0.135 (-0.170,-0.101)* |  |
| Mauritania | 461 (331,634) | 21.74 (15.44,30.36) | 758 (529,1061) | 17.46 (12.08,25.06) | 64.6% (48.6%,82%) | -0.643 (-0.746,-0.540)* |  |
| Mauritius | 213 (146,307) | 18.73 (12.86,26.98) | 228 (151,342) | 17.82 (11.83,26.57) | 6.9% (-5.7%,20.9%) | -0.145 (-0.388,0.098) |  |
| Mexico | 52580 (32983,78596) | 58.76 (37.34,88.81) | 48577 (31039,73639) | 37.8 (24.21,57.38) | -7.6% (-12.4%,-2%) | -1.341 (-1.605,-1.076)* |  |
| Micronesia (Federated States of) | 21 (14,29) | 20.79 (14.31,29.68) | 21 (14,31) | 21.11 (14.14,30.81) | 4.2% (-4.6%,13.8%) | 0.101 (-0.331,0.535) |  |
| Monaco | 8 (5,12) | 27.07 (17.37,39.27) | 10 (6,15) | 25.72 (16.16,38.28) | 19.8% (14.3%,25.1%) | -0.160 (-0.189,-0.131)* |  |
| Mongolia | 1062 (724,1526) | 46.09 (31.34,66.75) | 1708 (1115,2540) | 50.1 (32.61,74.62) | 60.9% (45.7%,78.6%) | 0.330 (0.144,0.517)* |  |
| Montenegro | 481 (305,741) | 76.63 (48.84,117.78) | 382 (239,603) | 64.95 (40.88,101.05) | -20.5% (-24.1%,-17%) | -0.532 (-0.586,-0.478)* |  |
| Morocco | 10250 (7068,14410) | 38.92 (26.73,54.4) | 11896 (8004,17293) | 31.89 (21.38,46.54) | 16.1% (5.2%,31%) | -0.649 (-0.778,-0.521)* |  |
| Mozambique | 5148 (3138,9049) | 34.58 (21.49,59.72) | 6818 (4800,9284) | 21.36 (15.03,29.15) | 32.5% (-10.1%,74.8%) | -1.562 (-2.577,-0.536)* |  |
| Myanmar | 13451 (9364,18691) | 32.2 (22.41,45.11) | 25794 (17871,37867) | 45.01 (31.19,65.81) | 91.8% (49.7%,164.6%) | -0.201 (-1.018,0.623) |  |
| Namibia | 284 (204,389) | 19.88 (14.33,27.32) | 483 (343,669) | 19.53 (13.86,27.33) | 70.2% (58.5%,82.5%) | -0.070 (-0.327,0.188) |  |
| Nauru | 2 (2,3) | 22.66 (15.47,32.55) | 3 (2,4) | 24.25 (16.14,35.56) | 16.3% (7.5%,25.2%) | 0.215 (0.193,0.237)* |  |
| Nepal | 5759 (3759,8815) | 30.26 (19.49,46.73) | 9940 (6061,16287) | 31.92 (19.74,52.83) | 72.6% (53.6%,90.5%) | 0.115 (-1.311,1.563) |  |
| Netherlands | 4038 (2638,5796) | 26.51 (17.36,37.71) | 5223 (3137,8304) | 25.67 (15.96,38.75) | 29.3% (10.9%,51.4%) | -0.003 (-0.270,0.264) |  |
| New Zealand | 2249 (1434,3303) | 65.9 (41.7,96.2) | 2869 (1814,4209) | 57 (35.85,83.58) | 27.6% (17.7%,37.1%) | -0.453 (-0.533,-0.373)* |  |
| Nicaragua | 1634 (1134,2394) | 36.07 (24.45,52.31) | 1989 (1322,3000) | 30.07 (19.9,46.01) | 21.7% (8.8%,35.6%) | -0.479 (-0.935,-0.021)* |  |
| Niger | 2046 (1479,2775) | 23.02 (16.55,31.3) | 6246 (4390,8586) | 23.32 (16.71,31.52) | 205.3% (180.6%,242.7%) | 0.382 (-0.081,0.848) |  |
| Nigeria | 18574 (13228,25958) | 19.55 (13.7,27.52) | 44214 (31526,60116) | 18.86 (13.28,25.94) | 138.1% (116.2%,172.7%) | -0.103 (-1.159,0.963) |  |
| Niue | 0 (0,1) | 19.77 (13.29,28.56) | 0 (0,0) | 19 (12.53,28.63) | -29.3% (-35.8%,-22.4%) | -0.254 (-0.711,0.206) |  |
| North Macedonia | 1328 (876,1951) | 66.35 (43.84,97.07) | 1198 (768,1857) | 59.14 (37.72,92.12) | -9.8% (-23.4%,5.9%) | -0.464 (-0.874,-0.053)* |  |
| Northern Mariana Islands | 13 (9,19) | 28.35 (18.54,40.85) | 13 (8,19) | 26.56 (17.53,39.76) | -3.9% (-15.5%,7.8%) | -0.223 (-0.421,-0.024)* |  |
| Norway | 1651 (1061,2471) | 36.37 (23.23,52.31) | 1690 (1016,2691) | 27.77 (17.19,42.19) | 2.4% (-8.2%,12.1%) | -0.860 (-1.339,-0.379)* |  |
| Oman | 1059 (695,1539) | 52.94 (34.99,78.1) | 1840 (1218,2661) | 39.76 (26.49,58.2) | 73.8% (49.3%,104.4%) | -0.919 (-1.002,-0.836)* |  |
| Pakistan | 20680 (14436,28943) | 18.46 (12.69,25.79) | 41087 (28924,56785) | 17.27 (12.1,23.73) | 98.7% (87.4%,115.1%) | -0.728 (-2.212,0.778) |  |
| Palau | 5 (3,7) | 31.32 (20.58,46.62) | 6 (4,9) | 33.53 (21.46,51.39) | 27.3% (14.4%,41.2%) | 0.221 (0.199,0.242)* |  |
| Palestine | 1327 (826,2292) | 57.62 (36.83,97.94) | 1950 (1316,2757) | 35.49 (23.97,49.8) | 46.9% (9.5%,90.8%) | -1.878 (-7.174,3.721) |  |
| Panama | 933 (623,1378) | 36.67 (24.39,54.24) | 1312 (854,1956) | 30.88 (20.12,46.15) | 40.7% (32.9%,48.1%) | -0.573 (-0.749,-0.397)* |  |
| Papua New Guinea | 869 (592,1242) | 21.89 (14.88,31.83) | 2603 (1776,3706) | 26.26 (17.86,38.4) | 199.4% (175.2%,225.3%) | 0.007 (-0.544,0.562) |  |
| Paraguay | 1521 (992,2319) | 36.25 (23.66,54.94) | 2543 (1715,3671) | 34.58 (23.29,50.16) | 67.2% (55.9%,81.8%) | -0.152 (-0.227,-0.076)* |  |
| Peru | 11838 (8066,18445) | 49.01 (34.39,75.01) | 10397 (7038,15290) | 28.36 (19.25,41.62) | -12.2% (-47.5%,24.5%) | -1.943 (-2.237,-1.648)* |  |
| Philippines | 22445 (15871,30957) | 33.93 (24.15,46.76) | 22967 (15565,32096) | 20.16 (13.72,28.46) | 2.3% (-19.8%,21.6%) | -0.757 (-1.261,-0.250)* |  |
| Poland | 31447 (20523,47525) | 82.63 (54.15,124.57) | 24057 (14918,38306) | 62.92 (39.28,97.89) | -23.5% (-31.8%,-15.3%) | -0.873 (-0.936,-0.810)* |  |
| Portugal | 3717 (2501,5241) | 37 (24.87,52.01) | 2560 (1672,3730) | 22.65 (14.79,32.65) | -31.1% (-40.2%,-21.9%) | -1.578 (-1.657,-1.499)* |  |
| Puerto Rico | 1115 (748,1646) | 30.83 (20.7,45.43) | 1101 (716,1693) | 33.87 (22.23,49.84) | -1.3% (-15.3%,15%) | 0.333 (0.164,0.503)* |  |
| Qatar | 218 (145,311) | 43.22 (28.92,62.05) | 1253 (815,1862) | 37.37 (24.51,56.07) | 475% (408.9%,555.6%) | -0.464 (-0.519,-0.408)* |  |
| Republic of Korea | 21162 (13939,29305) | 47.31 (31.3,65.73) | 16011 (10203,23898) | 28.87 (18.39,42.33) | -24.3% (-34.6%,-10.6%) | -1.574 (-1.696,-1.452)* |  |
| Republic of Moldova | 3258 (2127,4785) | 73.09 (47.77,107.42) | 1585 (1041,2386) | 46.54 (30.46,69.87) | -51.4% (-54.8%,-48%) | -1.820 (-2.008,-1.632)* |  |
| Romania | 20763 (13153,31615) | 91 (57.79,137.3) | 11877 (7374,18452) | 67.8 (42.83,104.39) | -42.8% (-45.8%,-39.1%) | -0.937 (-1.005,-0.869)* |  |
| Russian Federation | 127097 (82072,187430) | 85.45 (55.7,125.25) | 91905 (58992,138832) | 65.26 (42.49,98.55) | -27.7% (-34.7%,-19.8%) | -0.869 (-1.118,-0.619)* |  |
| Rwanda | 4941 (2805,9338) | 60.31 (35.3,111.92) | 2153 (1472,3044) | 16.47 (11.36,23.51) | -56.4% (-78.8%,-23.8%) | -3.325 (-4.913,-1.710)* |  |
| Saint Kitts and Nevis | 13 (9,20) | 31.22 (21.31,46.84) | 18 (12,28) | 32.23 (21.38,49.24) | 37.5% (24.1%,52.3%) | 0.075 (-0.193,0.344) |  |
| Saint Lucia | 37 (26,53) | 26.28 (18.23,37.39) | 45 (30,67) | 26.71 (17.81,39.88) | 20.8% (9%,33.7%) | 0.043 (-0.127,0.213) |  |
| Saint Vincent and the Grenadines | 30 (21,43) | 26.1 (18.05,38.11) | 31 (21,47) | 28.04 (18.58,41.71) | 4.1% (-5.7%,14.7%) | 0.136 (-0.086,0.358) |  |
| Samoa | 40 (28,55) | 23.31 (16.32,31.56) | 39 (26,57) | 19.29 (12.85,28.33) | -1.5% (-28.4%,19.9%) | -0.206 (-0.860,0.451) |  |
| San Marino | 7 (4,10) | 28.9 (18.42,41.78) | 9 (6,14) | 27.27 (17.32,40.42) | 36.5% (24.7%,48%) | -0.187 (-0.215,-0.159)* |  |
| Sao Tome and Principe | 29 (20,41) | 21.41 (14.65,31.31) | 58 (40,82) | 27.38 (18.77,39.57) | 102.1% (76.5%,146.5%) | 0.759 (0.592,0.927)* |  |
| Saudi Arabia | 10562 (6736,16029) | 67.55 (43.01,101.38) | 32300 (20100,50052) | 74.93 (46.9,114.87) | 205.8% (162.6%,243.6%) | 0.351 (0.155,0.548)* |  |
| Senegal | 1586 (1131,2208) | 19.01 (13.41,26.41) | 2613 (1801,3709) | 16.73 (11.53,24.12) | 64.7% (44.6%,85.3%) | -0.613 (-0.893,-0.333)* |  |
| Serbia | 6364 (4193,9609) | 68.61 (45.24,103.82) | 4916 (3051,7644) | 58.33 (36.64,90.88) | -22.8% (-30.3%,-13.8%) | -1.009 (-1.655,-0.360)* |  |
| Seychelles | 16 (11,24) | 22.44 (15.09,32.89) | 21 (14,30) | 19.35 (12.81,28.37) | 25.1% (11.2%,38.3%) | -0.583 (-0.837,-0.327)* |  |
| Sierra Leone | 852 (608,1177) | 18.26 (12.79,25.55) | 1520 (1069,2119) | 16.92 (11.76,24.23) | 78.4% (67.2%,91.2%) | -0.810 (-8.919,8.020) |  |
| Singapore | 997 (655,1433) | 31.4 (20.76,44.99) | 1377 (886,2014) | 25.48 (16.31,37.54) | 38.1% (26.5%,50.2%) | -0.670 (-0.822,-0.518)* |  |
| Slovakia | 4919 (3093,7551) | 92.47 (58.59,140.87) | 4120 (2505,6587) | 76.1 (47.02,119.59) | -16.2% (-22.9%,-9.6%) | -0.621 (-0.675,-0.566)* |  |
| Slovenia | 2228 (1394,3439) | 111.46 (69.96,171.92) | 1869 (1126,3051) | 84.43 (51.6,131.97) | -16.1% (-26%,-5%) | -0.910 (-1.060,-0.760)* |  |
| Solomon Islands | 82 (54,121) | 27.93 (18.34,41.89) | 201 (129,310) | 32.55 (20.54,51.32) | 145.6% (120.8%,171.5%) | 0.646 (0.310,0.984)* |  |
| Somalia | 5075 (2856,9635) | 57.12 (33.21,108.06) | 7253 (4939,11081) | 30.99 (21.42,46.38) | 42.9% (8%,86.7%) | -2.421 (-6.983,2.366) |  |
| South Africa | 12581 (8988,17848) | 32.63 (23.05,45.96) | 12682 (9046,17859) | 21.55 (15.44,30.43) | 0.8% (-5.7%,7.3%) | -1.357 (-1.503,-1.210)* |  |
| South Sudan | 1170 (832,1649) | 18.64 (13.26,26.18) | 2842 (2021,4124) | 26.66 (19.34,37.95) | 143% (88.5%,261.7%) | 2.236 (-6.862,12.224) |  |
| Spain | 12885 (8623,18211) | 33.08 (22.21,46.26) | 12108 (7635,18477) | 26.12 (16.48,39.46) | -6% (-19.8%,8.9%) | -0.783 (-0.994,-0.572)* |  |
| Sri Lanka | 19207 (10491,36953) | 101.59 (56.76,193.32) | 6552 (4239,10178) | 28.74 (18.74,44.57) | -65.9% (-85.2%,-33.7%) | -3.382 (-15.129,9.992) |  |
| Sudan | 14007 (8863,24913) | 63.18 (40.59,109.67) | 13001 (8866,18376) | 28.62 (19.52,40.63) | -7.2% (-45.6%,38.1%) | -2.508 (-5.993,1.107) |  |
| Suriname | 97 (68,138) | 23.93 (16.84,34.04) | 143 (97,209) | 25.15 (17.14,36.61) | 47.2% (37.7%,59.4%) | 0.179 (0.109,0.248)* |  |
| Sweden | 2993 (1890,4473) | 33.35 (20.98,49.18) | 3155 (1942,4948) | 27.08 (16.93,40.48) | 5.4% (-2.8%,12.7%) | -0.675 (-0.760,-0.590)* |  |
| Switzerland | 3831 (2397,5696) | 50.84 (31.82,75.82) | 3286 (1959,5252) | 33.47 (20.36,51.92) | -14.2% (-25.9%,-4%) | -1.365 (-1.496,-1.234)* |  |
| Syrian Arab Republic | 3823 (2579,5459) | 27.81 (18.64,39.7) | 5823 (3797,8831) | 40.8 (26.66,61.61) | 52.3% (17.9%,132%) | 1.351 (-1.305,4.078) |  |
| Taiwan (Province of China) | 6090 (4078,8908) | 29.56 (19.81,43.21) | 4216 (2695,6455) | 15.83 (10.48,23.49) | -30.8% (-38.6%,-22.6%) | -2.533 (-2.892,-2.173)* |  |
| Tajikistan | 2414 (1635,3456) | 41.61 (27.89,60.56) | 3256 (2181,4734) | 30.51 (20.5,44.57) | 34.9% (27.9%,42.2%) | -2.329 (-3.296,-1.353)* |  |
| Thailand | 18212 (12425,25977) | 30.79 (20.84,43.8) | 18136 (12118,26632) | 26.53 (17.73,38.82) | -0.4% (-17.5%,21.5%) | -0.608 (-0.889,-0.327)* |  |
| Timor-Leste | 688 (367,1337) | 79.09 (43.25,151.82) | 285 (201,389) | 20.38 (14.28,28.31) | -58.6% (-80.8%,-22.1%) | -5.158 (-8.184,-2.033)* |  |
| Togo | 752 (535,1044) | 19.51 (13.5,27.31) | 1478 (1036,2065) | 18.1 (12.61,25.74) | 96.4% (84.2%,110.5%) | -0.371 (-0.690,-0.051)* |  |
| Tokelau | 0 (0,0) | 18.17 (12.15,26.02) | 0 (0,0) | 17.39 (11.39,26.11) | -15.3% (-24.8%,-3.6%) | -0.138 (-0.185,-0.090)* |  |
| Tonga | 17 (12,24) | 17.66 (12.16,24.82) | 15 (10,22) | 14.56 (9.63,21.41) | -11.7% (-22.8%,-1.8%) | -0.444 (-0.785,-0.102)* |  |
| Trinidad and Tobago | 361 (258,502) | 29.02 (20.68,40.37) | 383 (275,541) | 29.84 (21.66,42.25) | 6.3% (-1.6%,17.2%) | 0.217 (-0.693,1.135) |  |
| Tunisia | 3210 (2147,4624) | 36.63 (24.72,52.71) | 3690 (2427,5435) | 31.53 (20.78,46.36) | 14.9% (3%,28.6%) | -0.408 (-0.529,-0.286)* |  |
| Turkmenistan | 1603 (1079,2319) | 38.7 (26.23,56.24) | 1703 (1140,2537) | 32.1 (21.45,47.83) | 6.3% (-3.9%,18.2%) | -0.855 (-1.580,-0.124)* |  |
| Tuvalu | 2 (1,3) | 20.54 (14.05,29.24) | 2 (2,4) | 19.77 (13.26,28.7) | 26.4% (13.3%,41.1%) | -0.175 (-0.290,-0.059)* |  |
| T眉rkiye | 18578 (12832,25949) | 30.77 (21.27,43.01) | 21808 (14154,32856) | 26.36 (17.18,39.89) | 17.4% (-0.6%,40.3%) | -0.220 (-1.285,0.857) |  |
| Uganda | 4448 (3155,6332) | 23.05 (16.38,32.07) | 7610 (5419,10364) | 17.19 (12.04,24.05) | 71.1% (23.6%,107%) | -1.091 (-3.648,1.533) |  |
| Ukraine | 42543 (27356,63238) | 83.12 (53.58,123.42) | 27988 (18246,41955) | 68.79 (45.07,102.76) | -34.2% (-37.5%,-31%) | -0.613 (-1.485,0.267) |  |
| United Arab Emirates | 852 (568,1234) | 42.28 (28.28,61.18) | 4177 (2767,6090) | 38.55 (25.71,56.95) | 390.4% (343.2%,442.9%) | -0.333 (-0.408,-0.259)* |  |
| United Kingdom | 17432 (11235,24704) | 29.82 (19.22,42.59) | 17571 (10765,27356) | 24.38 (15.26,36.52) | 0.8% (-9.5%,11.5%) | -0.656 (-0.816,-0.495)* |  |
| United Republic of Tanzania | 5289 (3769,7256) | 18.83 (13.15,26.13) | 10163 (6983,14340) | 17.14 (11.71,24.65) | 92.2% (79.7%,104.4%) | -0.253 (-0.552,0.047) |  |
| United States of America | 82320 (54789,112967) | 31.78 (21.02,43.64) | 100249 (65951,144601) | 26.53 (17.88,37.02) | 21.8% (5.7%,38.8%) | -0.612 (-1.022,-0.200)* |  |
| United States Virgin Islands | 30 (21,43) | 28.44 (19.64,41.12) | 22 (15,33) | 26.27 (17.74,39.1) | -26.1% (-34.7%,-15%) | -0.285 (-0.583,0.013) |  |
| Uruguay | 1384 (848,2219) | 44.8 (27.32,71.83) | 1217 (758,1893) | 36.7 (22.83,56.44) | -12.1% (-20.4%,-1.4%) | -0.649 (-0.716,-0.582)* |  |
| Uzbekistan | 9171 (6172,13283) | 40.37 (27.49,58.45) | 11635 (7869,16837) | 33.44 (22.52,48.45) | 26.9% (21.4%,32.9%) | -0.624 (-0.783,-0.464)* |  |
| Vanuatu | 26 (18,37) | 17.85 (12.04,25.28) | 53 (36,76) | 17.44 (11.79,24.93) | 101.7% (89.7%,113.2%) | -0.141 (-0.541,0.261) |  |
| Venezuela (Bolivarian Republic of) | 9672 (6350,14246) | 47.1 (30.95,69.8) | 10376 (6722,15577) | 41.03 (26.54,61.26) | 7.3% (1.1%,14.9%) | -0.608 (-1.010,-0.204)* |  |
| Viet Nam | 17047 (11679,24779) | 26.1 (17.88,37.55) | 27730 (18128,41337) | 28.07 (18.32,42.22) | 62.7% (37.7%,91.7%) | 0.279 (0.043,0.515)* |  |
| Yemen | 4910 (3305,7003) | 34.01 (23.11,48.16) | 35253 (19523,69397) | 93.92 (53.04,182.95) | 618% (304.9%,1438.9%) | 3.129 (-0.215,6.584) |  |
| Zambia | 1613 (1152,2237) | 19.02 (13.31,26.31) | 3335 (2318,4758) | 17.93 (12.37,25.7) | 106.8% (90.1%,125.1%) | -0.110 (-0.250,0.031) |  |
| Zimbabwe | 1928 (1356,2629) | 18.95 (13.53,26.46) | 2682 (1889,3741) | 17.61 (12.42,24.64) | 39.1% (32.9%,45.5%) | -0.212 (-0.377,-0.047)* |  |

AAPC: average annual percent change; ASIR: age-standardized incidence rate; CI: Confidence interval; GBD: global Burden of Disease; SDI: sociodemographic index; UI: Uncertainty intervals (**P* < 0.05)

**S5 Table.** **YLDs and ASYR of hip dislocation in 1990 and 2021, and the AAPC values for 1990 to 2021, for 204 countries and regions**

| **Countries or territories** | **1990** | | **2021** | | **1990–2021** | |  |
| --- | --- | --- | --- | --- | --- | --- | --- |
|  | **YLDs** | **ASYR per 100,000** | **YLDs** | **ASYR per 100,000** | **Change of number** | **AAPC** |  |
|  |  |  |  |  |  |  |  |
|  | **n (95% UI)** | **n (95% UI)** | **n (95% UI)** | **n (95% UI)** | **n (95% UI)** | **n (95% CI)** |  |
| Afghanistan | 845 (164,2932) | 10.25 (2.04,34.92) | 1796 (578,4540) | 9.03 (2.61,24.67) | 112.7% (34.3%,408.2%) | -0.396 (-0.509,-0.283)* |  |
| Albania | 56 (28,96) | 2.03 (1.03,3.49) | 62 (31,107) | 1.83 (0.92,3.15) | 10.7% (-1%,26.2%) | -0.358 (-0.465,-0.252)* |  |
| Algeria | 276 (145,485) | 1.57 (0.84,2.72) | 506 (257,887) | 1.19 (0.61,2.1) | 83.5% (53.5%,132.3%) | -0.876 (-0.944,-0.809)* |  |
| American Samoa | 0 (0,0) | 0.8 (0.42,1.42) | 0 (0,1) | 0.92 (0.47,1.68) | 72% (40.5%,128.4%) | 0.434 (0.308,0.560)* |  |
| Andorra | 1 (1,2) | 1.69 (0.89,2.92) | 2 (1,4) | 1.68 (0.88,2.95) | 133.4% (108.3%,161%) | -0.011 (-0.058,0.036) |  |
| Angola | 213 (87,485) | 2.34 (0.99,4.99) | 397 (166,859) | 2.06 (0.87,4.5) | 85.8% (65.8%,118.7%) | -0.336 (-0.829,0.160) |  |
| Antigua and Barbuda | 0 (0,1) | 0.83 (0.43,1.46) | 1 (0,2) | 0.86 (0.45,1.48) | 87.6% (62.6%,118.5%) | 0.021 (-0.157,0.198) |  |
| Argentina | 444 (232,770) | 1.38 (0.72,2.39) | 642 (330,1098) | 1.24 (0.64,2.11) | 44.7% (31.2%,60.6%) | -0.348 (-0.392,-0.304)* |  |
| Armenia | 89 (42,189) | 2.8 (1.32,5.9) | 64 (34,115) | 1.85 (0.95,3.42) | -28.3% (-42.2%,0.5%) | -1.333 (-1.719,-0.946)* |  |
| Australia | 414 (216,708) | 2.23 (1.16,3.84) | 694 (368,1210) | 1.86 (0.98,3.23) | 67.7% (54.2%,84%) | -0.581 (-0.620,-0.542)* |  |
| Austria | 186 (97,322) | 1.84 (0.95,3.18) | 197 (104,350) | 1.35 (0.71,2.39) | 6% (-4.4%,17%) | -0.980 (-1.046,-0.914)* |  |
| Azerbaijan | 79 (41,140) | 1.26 (0.66,2.18) | 131 (68,229) | 1.17 (0.6,2.05) | 65.3% (31.7%,125.9%) | -0.194 (-0.519,0.133) |  |
| Bahamas | 2 (1,3) | 0.9 (0.47,1.57) | 4 (2,7) | 0.99 (0.53,1.76) | 118.4% (81.9%,164.9%) | 0.353 (0.182,0.525)* |  |
| Bahrain | 4 (2,7) | 1 (0.53,1.72) | 13 (7,23) | 0.84 (0.43,1.46) | 236.6% (183.9%,308.7%) | -0.577 (-0.699,-0.454)* |  |
| Bangladesh | 482 (243,894) | 0.61 (0.31,1.1) | 1067 (525,1983) | 0.67 (0.33,1.25) | 121.1% (75.7%,177.3%) | 0.152 (-0.116,0.420) |  |
| Barbados | 2 (1,3) | 0.71 (0.37,1.25) | 3 (1,5) | 0.71 (0.37,1.23) | 50% (30.5%,73.7%) | -0.033 (-0.104,0.039) |  |
| Belarus | 275 (139,480) | 2.32 (1.16,4.04) | 277 (141,495) | 2.09 (1.06,3.76) | 0.6% (-8.8%,12%) | -0.322 (-0.401,-0.243)* |  |
| Belgium | 237 (120,417) | 1.8 (0.9,3.15) | 309 (167,529) | 1.6 (0.86,2.75) | 30.4% (19.2%,44.6%) | -0.361 (-0.434,-0.287)* |  |
| Belize | 1 (1,2) | 0.92 (0.48,1.6) | 4 (2,7) | 1.09 (0.58,1.88) | 239.4% (185.6%,296.9%) | 0.549 (0.444,0.655)* |  |
| Benin | 21 (11,38) | 0.7 (0.36,1.22) | 55 (29,95) | 0.66 (0.34,1.12) | 159.5% (119.2%,209.3%) | -0.194 (-0.251,-0.138)* |  |
| Bermuda | 1 (0,1) | 0.78 (0.41,1.35) | 1 (0,1) | 0.7 (0.36,1.25) | 38.7% (20.4%,59.3%) | -0.348 (-0.412,-0.285)* |  |
| Bhutan | 3 (1,5) | 0.7 (0.36,1.22) | 6 (3,11) | 0.89 (0.46,1.56) | 126.1% (87.8%,172.8%) | 0.798 (0.665,0.932)* |  |
| Bolivia (Plurinational State of) | 51 (27,89) | 1.11 (0.58,1.9) | 96 (50,165) | 0.91 (0.47,1.57) | 87.6% (62.8%,117.6%) | -0.639 (-0.679,-0.599)* |  |
| Bosnia and Herzegovina | 96 (49,167) | 2.11 (1.08,3.7) | 125 (63,226) | 2.76 (1.41,5.06) | 29.9% (-4.2%,97.2%) | 1.006 (0.738,1.276)* |  |
| Botswana | 6 (3,11) | 0.75 (0.39,1.33) | 17 (9,30) | 0.84 (0.45,1.45) | 174.2% (134.9%,225.7%) | 0.348 (0.239,0.457)* |  |
| Brazil | 2131 (1122,3723) | 1.83 (0.96,3.17) | 3632 (1964,6242) | 1.48 (0.8,2.53) | 70.4% (58.4%,89.8%) | -0.684 (-0.724,-0.645)* |  |
| Brunei Darussalam | 3 (2,5) | 1.76 (0.92,3) | 5 (3,10) | 1.26 (0.66,2.2) | 76.7% (54.6%,102.3%) | -1.068 (-1.136,-1.000)* |  |
| Bulgaria | 258 (134,455) | 2.45 (1.28,4.31) | 197 (102,356) | 1.97 (1.02,3.53) | -23.6% (-30.2%,-16%) | -0.704 (-0.760,-0.649)* |  |
| Burkina Faso | 45 (24,79) | 0.75 (0.39,1.31) | 118 (62,212) | 0.79 (0.41,1.37) | 160.9% (117.6%,213.6%) | 0.179 (0.014,0.345)* |  |
| Burundi | 24 (13,42) | 0.69 (0.37,1.19) | 489 (152,1236) | 5.05 (1.6,12.67) | 1906.3% (740.7%,4305.6%) | 6.659 (6.409,6.910)* |  |
| Cabo Verde | 2 (1,3) | 0.74 (0.39,1.31) | 4 (2,6) | 0.71 (0.36,1.2) | 92.1% (64.1%,122.5%) | -0.144 (-0.187,-0.101)* |  |
| Cambodia | 323 (85,1020) | 3.52 (1.07,10.4) | 359 (138,924) | 2.43 (0.96,6.21) | 11% (-9.8%,75.4%) | -1.182 (-1.229,-1.135)* |  |
| Cameroon | 47 (24,82) | 0.71 (0.36,1.21) | 166 (84,292) | 0.77 (0.4,1.32) | 249.6% (178.2%,359.5%) | 0.268 (0.205,0.331)* |  |
| Canada | 446 (230,793) | 1.42 (0.74,2.52) | 710 (379,1201) | 1.15 (0.6,1.91) | 59.1% (42.9%,75.4%) | -0.694 (-0.732,-0.655)* |  |
| Central African Republic | 15 (8,26) | 0.84 (0.44,1.45) | 67 (31,132) | 1.48 (0.7,2.85) | 350.2% (194.2%,680%) | 1.852 (1.569,2.137)* |  |
| Chad | 66 (27,139) | 1.34 (0.58,2.72) | 130 (61,257) | 1.32 (0.62,2.59) | 97.7% (67.2%,150.6%) | -0.047 (-0.230,0.136) |  |
| Chile | 156 (83,272) | 1.35 (0.71,2.36) | 281 (149,492) | 1.23 (0.64,2.13) | 80.1% (60.1%,105.4%) | -0.329 (-0.416,-0.242)* |  |
| China | 12079 (6374,20796) | 1.2 (0.63,2.05) | 21072 (11095,36416) | 1.11 (0.59,1.93) | 74.4% (64.7%,84.7%) | -0.237 (-0.449,-0.024)* |  |
| Colombia | 498 (264,871) | 1.89 (0.99,3.26) | 740 (393,1311) | 1.38 (0.73,2.45) | 48.5% (27.8%,76.8%) | -1.035 (-1.098,-0.972)* |  |
| Comoros | 2 (1,4) | 0.71 (0.37,1.22) | 4 (2,8) | 0.71 (0.36,1.26) | 107.1% (70.1%,155.6%) | 0.061 (-0.211,0.334) |  |
| Congo | 12 (6,22) | 0.8 (0.41,1.43) | 67 (29,142) | 1.48 (0.66,2.99) | 442.1% (215.3%,928.3%) | 2.051 (1.818,2.284)* |  |
| Cook Islands | 0 (0,0) | 0.9 (0.48,1.58) | 0 (0,0) | 0.87 (0.45,1.54) | 37.4% (18%,61.3%) | -0.137 (-0.408,0.135) |  |
| Costa Rica | 28 (14,48) | 1.2 (0.62,2.09) | 56 (30,97) | 1.06 (0.56,1.84) | 103.8% (79.7%,132.6%) | -0.387 (-0.423,-0.351)* |  |
| Croatia | 136 (72,237) | 2.43 (1.28,4.24) | 157 (82,262) | 2.27 (1.2,3.81) | 15% (0.8%,40%) | -0.241 (-0.339,-0.142)* |  |
| Cuba | 125 (66,218) | 1.17 (0.61,2.04) | 200 (106,338) | 1.17 (0.62,2.01) | 60% (41.6%,80.3%) | -0.017 (-0.073,0.038) |  |
| Cyprus | 13 (7,23) | 1.71 (0.89,2.96) | 24 (12,42) | 1.33 (0.69,2.34) | 80.3% (63.7%,98.9%) | -0.807 (-0.841,-0.773)* |  |
| Czechia | 359 (186,621) | 2.92 (1.51,5.08) | 317 (166,551) | 1.95 (1.01,3.43) | -11.7% (-19.1%,-4%) | -1.308 (-1.393,-1.224)* |  |
| C么te d'Ivoire | 50 (25,90) | 0.7 (0.37,1.21) | 135 (71,231) | 0.73 (0.39,1.24) | 167.9% (118.7%,234%) | 0.127 (0.028,0.227)* |  |
| Democratic People's Republic of Korea | 169 (89,294) | 0.9 (0.48,1.57) | 242 (127,418) | 0.76 (0.4,1.31) | 43.7% (25.4%,61.8%) | -0.569 (-0.619,-0.518)* |  |
| Democratic Republic of the Congo | 195 (101,335) | 0.79 (0.41,1.35) | 705 (332,1323) | 1.11 (0.53,2.1) | 260.9% (147%,499%) | 1.134 (0.781,1.488)* |  |
| Denmark | 123 (65,218) | 1.71 (0.89,3.03) | 104 (55,178) | 1.13 (0.58,1.92) | -15.9% (-24.4%,-6.7%) | -1.339 (-1.415,-1.263)* |  |
| Djibouti | 2 (1,3) | 0.7 (0.37,1.24) | 8 (4,14) | 0.78 (0.41,1.41) | 299.6% (218.2%,411.5%) | 0.306 (0.144,0.469)* |  |
| Dominica | 0 (0,1) | 0.79 (0.41,1.32) | 1 (0,1) | 0.9 (0.47,1.67) | 37.7% (14.9%,68.6%) | 0.446 (0.334,0.558)* |  |
| Dominican Republic | 44 (23,78) | 0.82 (0.43,1.44) | 100 (50,180) | 0.93 (0.47,1.67) | 126.3% (92.9%,163.4%) | 0.383 (0.259,0.507)* |  |
| Ecuador | 94 (49,175) | 1.25 (0.65,2.3) | 206 (105,361) | 1.18 (0.6,2.07) | 117.8% (85.2%,156.1%) | -0.197 (-0.264,-0.129)* |  |
| Egypt | 535 (279,924) | 1.29 (0.68,2.19) | 895 (466,1522) | 1.05 (0.55,1.76) | 67.1% (41.9%,99.2%) | -0.691 (-0.795,-0.586)* |  |
| El Salvador | 272 (104,635) | 5.3 (2.2,11.63) | 198 (90,408) | 3.24 (1.45,6.74) | -27% (-36.8%,-5.8%) | -1.580 (-1.616,-1.544)* |  |
| Equatorial Guinea | 2 (1,4) | 0.8 (0.42,1.38) | 6 (3,10) | 0.61 (0.32,1.04) | 154.4% (109.6%,208.1%) | -0.837 (-0.896,-0.778)* |  |
| Eritrea | 301 (92,774) | 8.83 (2.74,21.81) | 251 (80,640) | 5.84 (1.87,14.76) | -16.7% (-26%,7.8%) | -1.346 (-1.423,-1.270)* |  |
| Estonia | 53 (27,96) | 2.89 (1.47,5.19) | 31 (16,55) | 1.6 (0.8,2.86) | -41.3% (-47.6%,-35.3%) | -1.918 (-2.117,-1.719)* |  |
| Eswatini | 5 (2,8) | 0.94 (0.49,1.64) | 8 (4,14) | 0.91 (0.48,1.57) | 76.5% (52%,109.9%) | -0.084 (-0.173,0.006) |  |
| Ethiopia | 531 (261,1038) | 1.36 (0.69,2.58) | 846 (379,1826) | 1.16 (0.53,2.46) | 59.5% (24.1%,135.8%) | -0.738 (-1.118,-0.356)* |  |
| Fiji | 4 (2,7) | 0.67 (0.35,1.17) | 5 (3,10) | 0.63 (0.33,1.11) | 44.9% (23.3%,74.9%) | -0.170 (-0.244,-0.097)* |  |
| Finland | 145 (75,249) | 2.24 (1.16,3.88) | 158 (84,270) | 1.63 (0.86,2.8) | 9.2% (0.3%,19.2%) | -1.018 (-1.121,-0.915)* |  |
| France | 1502 (787,2551) | 2.02 (1.05,3.48) | 1753 (921,2995) | 1.53 (0.81,2.64) | 16.8% (6.2%,26.6%) | -0.881 (-0.940,-0.823)* |  |
| Gabon | 6 (3,10) | 0.81 (0.42,1.39) | 10 (5,18) | 0.7 (0.37,1.29) | 67.8% (44%,94.8%) | -0.448 (-0.494,-0.402)* |  |
| Gambia | 5 (2,10) | 0.77 (0.38,1.4) | 10 (5,18) | 0.7 (0.35,1.23) | 90.4% (40.5%,151%) | -0.323 (-0.372,-0.275)* |  |
| Georgia | 100 (51,172) | 1.69 (0.86,2.9) | 87 (45,151) | 1.88 (0.96,3.23) | -12.7% (-23.2%,1.4%) | 0.403 (0.283,0.524)* |  |
| Germany | 1648 (855,2841) | 1.52 (0.78,2.65) | 1844 (960,3082) | 1.22 (0.63,2.06) | 11.9% (1.9%,22.9%) | -0.696 (-0.739,-0.653)* |  |
| Ghana | 60 (32,104) | 0.64 (0.33,1.1) | 150 (78,269) | 0.63 (0.33,1.1) | 148.7% (108.8%,198.4%) | -0.017 (-0.073,0.040) |  |
| Greece | 214 (114,373) | 1.64 (0.85,2.87) | 204 (103,351) | 1.18 (0.6,2.03) | -4.7% (-14%,6.9%) | -1.055 (-1.114,-0.995)* |  |
| Greenland | 1 (0,2) | 2.09 (1.11,3.56) | 1 (1,2) | 1.57 (0.83,2.78) | 15.6% (3.2%,29.5%) | -0.936 (-1.005,-0.866)* |  |
| Grenada | 1 (1,2) | 1.44 (0.68,2.75) | 1 (1,2) | 1.15 (0.59,2.06) | 19.5% (1.1%,46.2%) | -0.722 (-0.772,-0.671)* |  |
| Guam | 1 (0,1) | 0.75 (0.39,1.33) | 1 (1,2) | 0.74 (0.38,1.29) | 69% (45.6%,98.8%) | -0.069 (-0.127,-0.011)* |  |
| Guatemala | 206 (89,448) | 3.12 (1.38,6.41) | 290 (141,546) | 2.25 (1.08,4.3) | 40.3% (13.2%,81.8%) | -1.054 (-1.098,-1.009)* |  |
| Guinea | 30 (15,53) | 0.7 (0.36,1.22) | 69 (36,121) | 0.77 (0.41,1.35) | 127.1% (85.5%,180.1%) | 0.305 (0.187,0.423)* |  |
| Guinea-Bissau | 6 (3,9) | 0.89 (0.45,1.55) | 12 (6,20) | 0.9 (0.46,1.52) | 107.4% (65%,176.5%) | -0.025 (-0.172,0.122) |  |
| Guyana | 6 (3,11) | 1.07 (0.54,1.87) | 8 (4,14) | 1.17 (0.63,2) | 38.2% (21.3%,58.6%) | 0.280 (0.226,0.334)* |  |
| Haiti | 52 (27,88) | 1.1 (0.58,1.87) | 329 (128,797) | 2.84 (1.14,6.86) | 533.3% (205.2%,1256.5%) | 3.109 (2.366,3.856)* |  |
| Honduras | 43 (22,73) | 1.32 (0.69,2.23) | 110 (58,200) | 1.3 (0.69,2.33) | 159.1% (116.3%,224.2%) | -0.142 (-0.486,0.203) |  |
| Hungary | 384 (199,664) | 2.97 (1.53,5.13) | 284 (150,504) | 1.86 (0.98,3.35) | -25.9% (-32.6%,-19.5%) | -1.495 (-1.666,-1.325)* |  |
| Iceland | 4 (2,7) | 1.53 (0.79,2.63) | 6 (3,10) | 1.23 (0.65,2.15) | 42.7% (27.8%,58.3%) | -0.684 (-0.813,-0.555)* |  |
| India | 7539 (3929,13072) | 1.29 (0.68,2.22) | 15230 (7897,26310) | 1.21 (0.64,2.09) | 102% (91.7%,114.5%) | -0.213 (-0.261,-0.164)* |  |
| Indonesia | 1586 (802,2897) | 1.11 (0.57,1.95) | 2245 (1167,4031) | 0.84 (0.44,1.5) | 41.6% (21.6%,68.7%) | -0.889 (-1.032,-0.745)* |  |
| Iran (Islamic Republic of) | 1129 (560,2111) | 2.55 (1.33,4.68) | 1441 (763,2532) | 1.6 (0.85,2.81) | 27.7% (11.3%,45.7%) | -1.489 (-1.562,-1.416)* |  |
| Iraq | 934 (348,2209) | 5.76 (2.25,12.99) | 1853 (772,4137) | 5.28 (2.21,11.73) | 98.3% (79%,128.7%) | -0.278 (-0.430,-0.125)* |  |
| Ireland | 52 (27,88) | 1.38 (0.71,2.35) | 80 (42,137) | 1.19 (0.61,2.07) | 52.7% (37.7%,68.9%) | -0.474 (-0.563,-0.384)* |  |
| Israel | 63 (32,107) | 1.33 (0.68,2.27) | 133 (71,221) | 1.24 (0.66,2.07) | 112.2% (85.4%,157%) | -0.219 (-0.477,0.039) |  |
| Italy | 1374 (720,2341) | 1.82 (0.94,3.1) | 1321 (695,2192) | 1.24 (0.64,2.09) | -3.8% (-8.3%,0.7%) | -1.225 (-1.268,-1.182)* |  |
| Jamaica | 16 (9,29) | 0.81 (0.42,1.4) | 25 (13,42) | 0.8 (0.43,1.37) | 51.2% (30.9%,76%) | -0.037 (-0.186,0.113) |  |
| Japan | 2108 (1086,3698) | 1.35 (0.7,2.39) | 2460 (1278,4181) | 0.97 (0.51,1.67) | 16.7% (10.1%,23.9%) | -1.046 (-1.084,-1.007)* |  |
| Jordan | 24 (13,43) | 1.01 (0.54,1.8) | 81 (42,147) | 0.77 (0.39,1.39) | 238.5% (187.5%,306.7%) | -0.904 (-0.976,-0.831)* |  |
| Kazakhstan | 269 (138,477) | 1.81 (0.93,3.2) | 286 (149,500) | 1.49 (0.77,2.61) | 6.6% (-4.5%,17.9%) | -0.630 (-0.718,-0.543)* |  |
| Kenya | 94 (50,166) | 0.69 (0.37,1.19) | 245 (130,424) | 0.71 (0.37,1.22) | 160.4% (138.5%,197.7%) | 0.062 (0.002,0.122)* |  |
| Kiribati | 0 (0,1) | 0.73 (0.39,1.27) | 1 (0,1) | 0.62 (0.33,1.09) | 56.5% (29.7%,91.3%) | -0.557 (-0.677,-0.436)* |  |
| Kuwait | 18 (9,32) | 1.32 (0.68,2.33) | 62 (33,109) | 1.19 (0.64,2.06) | 243.5% (167.8%,378.4%) | -0.505 (-0.689,-0.322)* |  |
| Kyrgyzstan | 58 (30,101) | 1.63 (0.84,2.83) | 65 (33,114) | 1.08 (0.54,1.88) | 11.6% (-2.4%,27.3%) | -1.328 (-1.441,-1.214)* |  |
| Lao People's Democratic Republic | 30 (15,52) | 1.01 (0.54,1.76) | 46 (24,80) | 0.76 (0.4,1.32) | 56% (32.9%,83.6%) | -0.921 (-0.953,-0.889)* |  |
| Latvia | 104 (53,181) | 3.24 (1.67,5.67) | 52 (26,92) | 1.78 (0.9,3.17) | -50.3% (-55.6%,-45.2%) | -1.951 (-2.091,-1.812)* |  |
| Lebanon | 151 (48,429) | 5.24 (1.74,14.65) | 163 (59,416) | 2.82 (1.01,7.27) | 7.6% (-4.4%,29.6%) | -1.989 (-2.046,-1.933)* |  |
| Lesotho | 9 (4,15) | 0.8 (0.41,1.4) | 16 (8,28) | 1.09 (0.58,1.87) | 84.3% (59.3%,109.8%) | 1.012 (0.926,1.098)* |  |
| Liberia | 20 (10,39) | 0.97 (0.5,1.77) | 81 (31,183) | 1.97 (0.8,4.4) | 304.1% (123%,555.6%) | 2.350 (2.073,2.628)* |  |
| Libya | 45 (23,80) | 1.46 (0.76,2.56) | 152 (78,277) | 2.14 (1.11,3.83) | 240.5% (164.7%,341.1%) | 1.307 (0.980,1.634)* |  |
| Lithuania | 121 (62,211) | 2.88 (1.48,5) | 84 (43,146) | 1.91 (0.98,3.33) | -30.8% (-37.4%,-23.1%) | -1.334 (-1.482,-1.185)* |  |
| Luxembourg | 11 (6,20) | 2.28 (1.18,3.95) | 14 (7,24) | 1.46 (0.76,2.61) | 21.7% (9.6%,34.4%) | -1.433 (-1.527,-1.338)* |  |
| Madagascar | 49 (25,89) | 0.62 (0.31,1.09) | 104 (52,180) | 0.54 (0.28,0.92) | 110.5% (73.4%,155.1%) | -0.449 (-0.525,-0.374)* |  |
| Malawi | 39 (20,67) | 0.61 (0.32,1.06) | 68 (35,119) | 0.56 (0.29,0.96) | 76.8% (47.5%,113%) | -0.316 (-0.405,-0.227)* |  |
| Malaysia | 100 (51,173) | 0.78 (0.41,1.34) | 225 (113,398) | 0.73 (0.37,1.29) | 126.2% (93.8%,161.7%) | -0.212 (-0.267,-0.156)* |  |
| Maldives | 1 (1,2) | 0.83 (0.44,1.44) | 4 (2,7) | 0.77 (0.4,1.36) | 227.4% (169.8%,305.2%) | -0.299 (-0.541,-0.056)* |  |
| Mali | 45 (23,79) | 0.79 (0.41,1.36) | 191 (90,377) | 1.25 (0.6,2.4) | 320.4% (184.9%,572.8%) | 1.437 (1.091,1.784)* |  |
| Malta | 6 (3,11) | 1.57 (0.82,2.71) | 9 (5,17) | 1.29 (0.68,2.25) | 47.5% (33.2%,64.4%) | -0.617 (-0.687,-0.546)* |  |
| Marshall Islands | 0 (0,0) | 0.87 (0.46,1.57) | 0 (0,1) | 0.81 (0.43,1.41) | 65.8% (44.1%,93.1%) | -0.220 (-0.328,-0.113)* |  |
| Mauritania | 11 (6,19) | 0.82 (0.41,1.38) | 20 (10,34) | 0.66 (0.35,1.13) | 73.8% (47.1%,105.1%) | -0.686 (-0.786,-0.586)* |  |
| Mauritius | 6 (3,11) | 0.65 (0.34,1.16) | 10 (5,18) | 0.62 (0.32,1.1) | 67.4% (41.2%,96.3%) | -0.159 (-0.262,-0.055)* |  |
| Mexico | 1206 (631,2108) | 1.98 (1.04,3.43) | 1670 (885,2874) | 1.27 (0.67,2.18) | 38.5% (31.5%,46.3%) | -1.420 (-1.585,-1.254)* |  |
| Micronesia (Federated States of) | 1 (0,1) | 0.93 (0.48,1.64) | 1 (0,1) | 0.96 (0.51,1.68) | 37.6% (18.8%,62.2%) | 0.099 (-0.004,0.202) |  |
| Monaco | 1 (0,1) | 1.21 (0.64,2.15) | 1 (0,1) | 1.13 (0.59,1.94) | 25.1% (13.8%,37.6%) | -0.215 (-0.256,-0.174)* |  |
| Mongolia | 24 (12,42) | 1.59 (0.83,2.78) | 50 (26,86) | 1.64 (0.86,2.83) | 105.7% (82.4%,132.2%) | 0.093 (0.027,0.159)* |  |
| Montenegro | 14 (7,24) | 2.16 (1.11,3.74) | 15 (8,26) | 1.81 (0.92,3.22) | 6.3% (-4%,17.2%) | -0.548 (-0.634,-0.462)* |  |
| Morocco | 263 (138,461) | 1.36 (0.71,2.36) | 398 (208,697) | 1.08 (0.57,1.89) | 51.7% (32%,74.6%) | -0.722 (-0.785,-0.658)* |  |
| Mozambique | 335 (118,837) | 2.77 (1.02,6.58) | 314 (137,700) | 1.88 (0.79,4.2) | -6.1% (-22.4%,33.5%) | -1.243 (-1.298,-1.188)* |  |
| Myanmar | 476 (214,1038) | 1.38 (0.65,2.82) | 881 (438,1688) | 1.61 (0.8,3.11) | 85.3% (30%,182.4%) | 0.330 (-0.121,0.783) |  |
| Namibia | 20 (9,41) | 1.65 (0.77,3.18) | 23 (12,41) | 1.22 (0.63,2.24) | 14.5% (-12.3%,58.2%) | -0.964 (-1.010,-0.919)* |  |
| Nauru | 0 (0,0) | 1.02 (0.53,1.8) | 0 (0,0) | 1.04 (0.55,1.8) | 18.9% (4.4%,36.4%) | 0.049 (-0.017,0.114) |  |
| Nepal | 136 (71,235) | 1.04 (0.55,1.8) | 402 (209,737) | 1.48 (0.77,2.69) | 196% (138.1%,285.2%) | 1.196 (0.876,1.517)* |  |
| Netherlands | 215 (112,368) | 1.17 (0.61,2.03) | 322 (172,561) | 1.11 (0.58,1.93) | 50.3% (35.7%,68.8%) | -0.135 (-0.222,-0.048)* |  |
| New Zealand | 94 (48,163) | 2.54 (1.3,4.41) | 144 (74,244) | 2.07 (1.08,3.51) | 53.5% (42.3%,66.1%) | -0.680 (-0.721,-0.640)* |  |
| Nicaragua | 180 (58,425) | 4.78 (1.71,10.82) | 169 (70,362) | 2.94 (1.21,6.33) | -6.6% (-18.5%,24.1%) | -1.566 (-1.650,-1.483)* |  |
| Niger | 38 (20,66) | 0.78 (0.42,1.33) | 118 (61,209) | 0.79 (0.41,1.36) | 210.2% (149.7%,290.3%) | 0.044 (-0.025,0.113) |  |
| Nigeria | 452 (236,781) | 0.73 (0.38,1.25) | 1130 (611,2007) | 0.74 (0.4,1.27) | 150.2% (118.7%,206.5%) | 0.036 (-0.018,0.090) |  |
| Niue | 0 (0,0) | 0.8 (0.42,1.39) | 0 (0,0) | 0.79 (0.41,1.35) | -11.5% (-23%,2.5%) | -0.149 (-0.385,0.088) |  |
| North Macedonia | 32 (16,57) | 1.61 (0.83,2.84) | 44 (22,80) | 1.57 (0.81,2.87) | 36.4% (22.5%,52.8%) | -0.075 (-0.163,0.013) |  |
| Northern Mariana Islands | 0 (0,1) | 1.04 (0.54,1.81) | 1 (0,1) | 1 (0.53,1.78) | 50.7% (28.9%,78.3%) | -0.144 (-0.221,-0.067)* |  |
| Norway | 95 (50,162) | 1.66 (0.87,2.86) | 100 (53,171) | 1.19 (0.63,2.06) | 5.5% (-1.3%,11.9%) | -1.059 (-1.127,-0.991)* |  |
| Oman | 27 (14,47) | 2.09 (1.12,3.61) | 56 (29,99) | 1.42 (0.73,2.46) | 107.9% (82.7%,141.5%) | -1.259 (-1.314,-1.204)* |  |
| Pakistan | 497 (259,871) | 0.65 (0.34,1.12) | 1677 (853,3113) | 0.85 (0.44,1.56) | 237.4% (156.5%,389.1%) | 0.845 (0.749,0.941)* |  |
| Palau | 0 (0,0) | 1.23 (0.64,2.17) | 0 (0,0) | 1.23 (0.65,2.17) | 82.3% (61.9%,109.8%) | 0.026 (-0.014,0.065) |  |
| Palestine | 70 (30,159) | 3.91 (1.7,8.64) | 183 (75,406) | 4.46 (1.84,9.73) | 160.2% (98.9%,210%) | 0.396 (0.168,0.625)* |  |
| Panama | 26 (13,47) | 1.28 (0.66,2.28) | 45 (23,81) | 1.01 (0.52,1.83) | 70.8% (41.1%,97.4%) | -0.753 (-0.801,-0.706)* |  |
| Papua New Guinea | 23 (12,41) | 0.89 (0.47,1.55) | 85 (44,153) | 1.15 (0.61,2.04) | 261.6% (202.9%,341.5%) | 0.764 (0.654,0.874)* |  |
| Paraguay | 34 (17,60) | 1.14 (0.59,2) | 73 (39,125) | 1.1 (0.59,1.88) | 114.1% (84.1%,150.2%) | -0.138 (-0.188,-0.088)* |  |
| Peru | 255 (124,477) | 1.35 (0.68,2.45) | 404 (207,726) | 1.12 (0.57,2.01) | 58% (35.2%,87.8%) | -0.612 (-0.695,-0.529)* |  |
| Philippines | 643 (316,1226) | 1.29 (0.66,2.38) | 1066 (546,1946) | 1.05 (0.54,1.92) | 65.7% (41.3%,91.1%) | -0.677 (-0.820,-0.535)* |  |
| Poland | 1025 (533,1777) | 2.47 (1.28,4.29) | 1036 (539,1809) | 1.82 (0.93,3.19) | 1.1% (-3.9%,6.4%) | -0.981 (-1.081,-0.881)* |  |
| Portugal | 223 (119,378) | 1.86 (0.99,3.18) | 197 (105,336) | 1.05 (0.55,1.82) | -11.5% (-20.8%,-1.9%) | -1.844 (-1.926,-1.762)* |  |
| Puerto Rico | 39 (20,68) | 1.07 (0.56,1.88) | 56 (30,101) | 1.08 (0.57,1.95) | 45.8% (25.8%,69.1%) | 0.011 (-0.087,0.109) |  |
| Qatar | 5 (3,9) | 1.47 (0.76,2.62) | 34 (17,62) | 1.16 (0.59,2.09) | 548.7% (438.1%,712%) | -0.735 (-0.803,-0.667)* |  |
| Republic of Korea | 842 (434,1517) | 2.28 (1.18,4.12) | 1033 (546,1803) | 1.27 (0.67,2.24) | 22.8% (9.3%,38.5%) | -1.863 (-1.915,-1.810)* |  |
| Republic of Moldova | 107 (57,186) | 2.39 (1.27,4.12) | 73 (37,128) | 1.46 (0.75,2.57) | -32.3% (-38.3%,-25.2%) | -1.574 (-1.681,-1.466)* |  |
| Romania | 703 (365,1231) | 2.71 (1.41,4.77) | 535 (265,940) | 1.93 (0.97,3.41) | -24% (-30.8%,-16.4%) | -1.092 (-1.163,-1.021)* |  |
| Russian Federation | 4699 (2443,8161) | 2.76 (1.44,4.79) | 4330 (2236,7470) | 2.19 (1.12,3.76) | -7.9% (-11.8%,-2.6%) | -0.734 (-0.897,-0.571)* |  |
| Rwanda | 43 (22,77) | 0.89 (0.46,1.57) | 564 (172,1419) | 5.61 (1.74,14.18) | 1197.4% (395.7%,2875.5%) | 6.110 (5.772,6.449)* |  |
| Saint Kitts and Nevis | 0 (0,1) | 1 (0.53,1.74) | 1 (0,1) | 0.97 (0.51,1.61) | 83.6% (61.9%,111.5%) | -0.096 (-0.195,0.004) |  |
| Saint Lucia | 1 (0,2) | 0.87 (0.46,1.51) | 2 (1,3) | 0.83 (0.43,1.46) | 98% (70.4%,129.6%) | -0.110 (-0.169,-0.051)* |  |
| Saint Vincent and the Grenadines | 1 (0,1) | 0.85 (0.44,1.5) | 1 (1,2) | 0.89 (0.47,1.55) | 59.7% (37.7%,83%) | 0.146 (0.054,0.238)* |  |
| Samoa | 1 (0,2) | 0.81 (0.43,1.44) | 2 (1,3) | 0.96 (0.5,1.78) | 82.1% (42.8%,149.7%) | 0.489 (0.336,0.641)* |  |
| San Marino | 0 (0,1) | 1.28 (0.68,2.21) | 1 (0,1) | 1.19 (0.62,2.1) | 75.5% (58.9%,94.5%) | -0.231 (-0.296,-0.165)* |  |
| Sao Tome and Principe | 1 (0,1) | 0.72 (0.37,1.24) | 1 (1,2) | 0.82 (0.43,1.41) | 106.7% (73.3%,143.6%) | 0.412 (0.345,0.479)* |  |
| Saudi Arabia | 220 (111,387) | 2.24 (1.15,3.9) | 875 (451,1582) | 2.56 (1.34,4.49) | 297.5% (252.9%,348.9%) | 0.439 (0.376,0.502)* |  |
| Senegal | 29 (15,51) | 0.6 (0.31,1.04) | 67 (35,118) | 0.62 (0.32,1.09) | 129% (90.8%,185.5%) | 0.117 (-0.038,0.272) |  |
| Serbia | 211 (109,363) | 1.98 (1.02,3.42) | 231 (123,399) | 1.87 (0.98,3.25) | 9.2% (-5.9%,35.2%) | -0.342 (-0.685,0.002) |  |
| Seychelles | 1 (0,1) | 0.83 (0.43,1.41) | 1 (0,1) | 0.71 (0.37,1.25) | 64.6% (41.1%,92.3%) | -0.464 (-0.518,-0.409)* |  |
| Sierra Leone | 19 (10,34) | 0.64 (0.33,1.11) | 93 (42,194) | 1.49 (0.68,3.06) | 389.4% (177.8%,793.5%) | 2.858 (2.590,3.127)* |  |
| Singapore | 37 (19,65) | 1.34 (0.68,2.31) | 83 (42,142) | 1.05 (0.54,1.81) | 121.7% (96.6%,149.2%) | -0.775 (-0.819,-0.732)* |  |
| Slovakia | 156 (79,270) | 2.74 (1.39,4.75) | 168 (86,292) | 2.15 (1.11,3.72) | 7.6% (-1.2%,17.8%) | -0.774 (-0.845,-0.702)* |  |
| Slovenia | 75 (39,129) | 3.25 (1.68,5.59) | 82 (43,143) | 2.35 (1.23,4.09) | 9.2% (0%,20.7%) | -1.022 (-1.117,-0.927)* |  |
| Solomon Islands | 2 (1,4) | 1.24 (0.65,2.2) | 6 (3,11) | 1.39 (0.72,2.47) | 164.4% (130.9%,204%) | 0.340 (0.247,0.432)* |  |
| Somalia | 74 (27,243) | 1.2 (0.5,3.31) | 267 (107,578) | 1.78 (0.74,4) | 262% (70.2%,619.9%) | 1.207 (0.997,1.418)* |  |
| South Africa | 461 (242,822) | 1.62 (0.86,2.82) | 557 (295,1008) | 1.01 (0.53,1.82) | 20.8% (13.2%,28.9%) | -1.514 (-1.565,-1.463)* |  |
| South Sudan | 55 (24,110) | 1.1 (0.51,2.1) | 130 (53,294) | 1.79 (0.76,4.01) | 137.7% (92.9%,183.4%) | 1.694 (1.426,1.963)* |  |
| Spain | 710 (368,1221) | 1.51 (0.77,2.6) | 884 (454,1514) | 1.16 (0.6,2.04) | 24.5% (12.1%,37.3%) | -0.835 (-0.908,-0.762)* |  |
| Sri Lanka | 259 (128,505) | 1.64 (0.83,3.1) | 683 (320,1432) | 2.79 (1.29,5.9) | 163.6% (102.5%,247%) | 1.694 (1.018,2.375)* |  |
| Sudan | 216 (109,394) | 1.41 (0.72,2.5) | 548 (266,1079) | 1.66 (0.83,3.15) | 153.8% (99.1%,215.8%) | 0.497 (0.287,0.707)* |  |
| Suriname | 4 (2,9) | 1.13 (0.56,2.2) | 6 (3,12) | 0.99 (0.5,1.82) | 56.2% (20.8%,94.5%) | -0.433 (-0.472,-0.393)* |  |
| Sweden | 180 (96,300) | 1.46 (0.77,2.46) | 198 (104,337) | 1.15 (0.6,1.97) | 9.6% (-0.4%,18.8%) | -0.783 (-0.893,-0.674)* |  |
| Switzerland | 222 (117,382) | 2.34 (1.22,4.04) | 208 (109,353) | 1.41 (0.74,2.44) | -6.3% (-14.4%,3.4%) | -1.628 (-1.713,-1.543)* |  |
| Syrian Arab Republic | 116 (55,238) | 1.26 (0.63,2.51) | 732 (276,1632) | 5.85 (2.17,12.88) | 534% (238.6%,1084.7%) | 5.173 (4.808,5.538)* |  |
| Taiwan (Province of China) | 215 (113,376) | 1.19 (0.63,2.08) | 247 (133,437) | 0.68 (0.37,1.21) | 14.8% (-0.2%,32.5%) | -1.825 (-1.885,-1.765)* |  |
| Tajikistan | 51 (27,91) | 1.34 (0.71,2.41) | 145 (72,280) | 1.72 (0.85,3.3) | 185.2% (90.3%,380.3%) | 1.115 (0.658,1.574)* |  |
| Thailand | 590 (312,1021) | 1.24 (0.65,2.16) | 962 (510,1693) | 1.03 (0.54,1.83) | 63.2% (42.6%,85.9%) | -0.604 (-0.710,-0.497)* |  |
| Timor-Leste | 31 (10,79) | 3.97 (1.44,9.62) | 42 (15,103) | 4.36 (1.47,10.77) | 36.7% (25.3%,51.3%) | 0.250 (0.020,0.481)* |  |
| Togo | 16 (8,28) | 0.73 (0.37,1.29) | 41 (21,73) | 0.71 (0.37,1.25) | 157.2% (116.7%,205.9%) | -0.091 (-0.200,0.018) |  |
| Tokelau | 0 (0,0) | 0.76 (0.39,1.3) | 0 (0,0) | 0.69 (0.36,1.2) | -3.6% (-16.2%,11.3%) | -0.310 (-0.426,-0.194)* |  |
| Tonga | 0 (0,1) | 0.66 (0.34,1.14) | 0 (0,1) | 0.56 (0.29,0.98) | 10.2% (-6.2%,29.3%) | -0.516 (-0.642,-0.390)* |  |
| Trinidad and Tobago | 9 (5,16) | 0.89 (0.46,1.55) | 16 (9,28) | 0.99 (0.53,1.7) | 79.5% (51.5%,112.1%) | 0.376 (0.301,0.451)* |  |
| Tunisia | 80 (42,146) | 1.23 (0.65,2.2) | 135 (73,239) | 1.03 (0.55,1.81) | 68.4% (49%,95%) | -0.567 (-0.638,-0.497)* |  |
| Turkmenistan | 35 (18,64) | 1.26 (0.65,2.25) | 49 (25,84) | 1 (0.52,1.71) | 38.2% (20%,61.6%) | -0.777 (-0.858,-0.696)* |  |
| Tuvalu | 0 (0,0) | 0.93 (0.49,1.61) | 0 (0,0) | 0.85 (0.44,1.45) | 30.2% (12.7%,52.2%) | -0.343 (-0.448,-0.238)* |  |
| T眉rkiye | 436 (227,781) | 0.94 (0.5,1.67) | 840 (443,1459) | 0.91 (0.48,1.59) | 92.5% (62%,132%) | -0.078 (-0.255,0.100) |  |
| Uganda | 331 (118,812) | 2.2 (0.85,5.03) | 357 (158,719) | 1.57 (0.67,3.31) | 8.1% (-13.3%,57.1%) | -1.084 (-1.172,-0.996)* |  |
| Ukraine | 1717 (901,3019) | 2.72 (1.42,4.8) | 1367 (718,2373) | 2.25 (1.18,3.92) | -20.4% (-26.6%,-12.8%) | -0.623 (-0.819,-0.427)* |  |
| United Arab Emirates | 21 (10,36) | 1.5 (0.77,2.6) | 156 (77,278) | 1.36 (0.7,2.46) | 642.8% (510.2%,804.3%) | -0.325 (-0.379,-0.272)* |  |
| United Kingdom | 1018 (529,1731) | 1.34 (0.7,2.3) | 1131 (593,1903) | 1.08 (0.56,1.82) | 11.1% (6.5%,15.9%) | -0.690 (-0.728,-0.652)* |  |
| United Republic of Tanzania | 112 (57,198) | 0.67 (0.35,1.2) | 245 (130,426) | 0.63 (0.34,1.09) | 118.7% (84.5%,161.7%) | -0.215 (-0.257,-0.172)* |  |
| United States of America | 4291 (2203,7433) | 1.46 (0.75,2.54) | 6471 (3451,11065) | 1.28 (0.68,2.2) | 50.8% (39.5%,61.7%) | -0.415 (-0.466,-0.363)* |  |
| United States Virgin Islands | 1 (1,2) | 1.01 (0.52,1.76) | 1 (1,2) | 0.85 (0.44,1.49) | 13.3% (-1.2%,32.1%) | -0.543 (-0.617,-0.469)* |  |
| Uruguay | 53 (27,91) | 1.54 (0.78,2.64) | 58 (29,99) | 1.3 (0.65,2.23) | 8.9% (-1.9%,19.5%) | -0.545 (-0.591,-0.498)* |  |
| Uzbekistan | 197 (103,346) | 1.26 (0.66,2.2) | 337 (177,607) | 1.05 (0.55,1.87) | 70.8% (48.3%,95.5%) | -0.591 (-0.645,-0.538)* |  |
| Vanuatu | 1 (0,1) | 0.8 (0.42,1.45) | 2 (1,3) | 0.79 (0.42,1.4) | 130.5% (97%,175.7%) | -0.082 (-0.203,0.039) |  |
| Venezuela (Bolivarian Republic of) | 218 (116,383) | 1.51 (0.8,2.6) | 424 (227,733) | 1.46 (0.78,2.51) | 94.2% (67.5%,123.2%) | -0.105 (-0.215,0.005) |  |
| Viet Nam | 436 (228,773) | 0.9 (0.47,1.58) | 1005 (537,1750) | 1 (0.54,1.73) | 130.2% (101.9%,162.3%) | 0.344 (0.298,0.391)* |  |
| Yemen | 144 (71,268) | 1.59 (0.8,2.87) | 690 (312,1443) | 2.38 (1.14,4.85) | 379.8% (233.3%,548.5%) | 1.361 (1.103,1.621)* |  |
| Zambia | 33 (17,59) | 0.69 (0.36,1.22) | 80 (42,139) | 0.68 (0.36,1.19) | 139.4% (97.7%,186.7%) | -0.057 (-0.114,0.001) |  |
| Zimbabwe | 47 (25,83) | 0.76 (0.4,1.33) | 76 (40,132) | 0.72 (0.38,1.23) | 60.1% (34.5%,93.1%) | -0.189 (-0.233,-0.144)* |  |

AAPC: average annual percent change; ASYR: age-standardized YLD rate; CI: Confidence interval; GBD: global Burden of Disease; SDI: sociodemographic index; UI: Uncertainty intervals (**P* < 0.05)

**S6 Table. Global incidence and years lived with disability for hip dislocation (all causes) in 2021 stratified by age group**

| **Age** | **Incidence** | **Years lived with disability** |
| --- | --- | --- |
| **<5** | 137108 (98114,184719) | 420 (211,784) |
| **5-9** | 167677 (114697,243574) | 1010 (531,1838) |
| **10-14** | 168360 (109349,250742) | 1736 (896,3151) |
| **15-19** | 231065 (155721,338003) | 2644 (1374,4926) |
| **20-24** | 224378 (151917,322800) | 4007 (2017,7202) |
| **25-29** | 194509 (128686,284409) | 5085 (2582,9366) |
| **30-34** | 191125 (123164,284519) | 6492 (3409,11700) |
| **35-39** | 172375 (111805,259639) | 7448 (3876,13395) |
| **40-44** | 140665 (91096,210627) | 7988 (4084,14546) |
| **45-49** | 126754 (77566,195678) | 8688 (4489,15768) |
| **50-54** | 119111 (71204,181103) | 9613 (4979,17240) |
| **55-59** | 109946 (68578,174521) | 9979 (5242,17466) |
| **60-64** | 96993 (58867,156909) | 9046 (4728,15960) |
| **65-69** | 91006 (53553,149100) | 9006 (4943,15742) |
| **70-74** | 78946 (44883,136832) | 7977 (4259,13874) |
| **75-79** | 65650 (35167,119909) | 6291 (3341,10548) |
| **80-84** | 57247 (31173,105369) | 5440 (2911,9212) |
| **85-89** | 36726 (20232,67363) | 3695 (1944,6347) |
| **90-94** | 15513 (8558,28211) | 1869 (984,3172) |
| **95+** | 4782 (2670,8642) | 713 (369,1199) |

**S7 Table. Global incidence and years lived with disability for hip dislocation (all causes) for all age groups in 1990 and 2021, and the age-standardized rates and AAPC values stratified by sex**

|  | | **n (95% UI)** | | | **ASR, per 100, 000** | | |
| --- | --- | --- | --- | --- | --- | --- | --- |
|  |  | **1990** | **2021** | **Change of number** | **1990 (95% UI)** | **2021 (95% UI)** | **AAPC (95% CI)** |
| **Incidence** | **Male** | 1207349 (852312,1633445) | 1369059 (952164,1892179) | 13.4% (5%,22.2%) | 44.01 (31.05,59.52) | 34.46 (24.07,47.65) | -0.788 (-1.115,-0.461)* |
|  | **Female** | 845575 (547873,1233377) | 1060877 (665645,1668246) | 25.5% (12.1%,40.6%) | 32.06 (20.87,47.21) | 26.45 (16.67,41.55) | -0.671 (-0.781,-0.562)* |
| **YLDs** | **Male** | 38136 (19461,67786) | 58398 (30818,101596) | 53.1% (46.5%,62.9%) | 1.69 (0.86,2.97 ) | 1.43 (0.75,2.48) | -0.547 (-0.600,-0.493)* |
|  | **Female** | 31294 (16358,54357) | 50748 (26828,87358) | 62.2% (54.6%,74.5%) | 1.36 (0.71,2.35) | 1.16 (0.61,2) | -0.487 (-0.567,-0.407)* |

AAPC: average annual percent change; ASR: age-standardized rate; UI: Uncertainty intervals; (*P < 0.05)

**S8 Table. Global age-standardized incidence and years lived with disability per 100,000 for hip dislocation stratified by the cause of injury in 1990 and 2021 and the AAPC values for 1990 to 2021**

| **Cause of injury** | **ASIR** | | | **ASYR** | | |  |
| --- | --- | --- | --- | --- | --- | --- | --- |
|  | **1990** | **2021** | **AAPC** | **1990** | **2021** | **AAPC** |  |
|  |  |  |  |  |  |  |  |
|  | **n (95% UI)** | **n (95% UI)** | **n(95% CI)** | **n (95% UI)** | **n (95% CI)** | **n(95% CI)** |  |
| **Environmental heat and cold exposure** | 0.39 (0.18,0.72) | 0.24 (0.11,0.45) | -1.559 (-1.586,-1.532)* | 0.02 (0.01,0.03) | 0.01 (0,0.02) | -1.768 (-1.822,-1.715)* |  |
| **Other transport injuries** | 0.35 (0.14,0.73) | 0.27 (0.11,0.56) | -0.857 (-0.937,-0.777)* | 0.03 (0.02,0.06) | 0.02 (0.01,0.04) | -0.838 (-1.110,-0.566)* |  |
| **Police conflict and executions** | 0.23 (0.09,0.5) | 0.38 (0.15,0.84) | 1.219 (-1.609,4.129) | 0.01 (0,0.01) | 0.02 (0,0.04) | 3.609 (3.461,3.758)* |  |
| **Foreign body** | 1.12 (0.41,2.4) | 0.94 (0.33,2.05) | -0.556 (-0.588,-0.525)* | 0.06 (0.03,0.1) | 0.04 (0.02,0.08) | -0.855 (-0.969,-0.741)* |  |
| **Interpersonal violence** | 1.73 (0.7,3.86) | 1.18 (0.48,2.61) | -1.240 (-1.310,-1.170)* | 0.11 (0.06,0.19) | 0.07 (0.04,0.12) | -1.536 (-1.614,-1.458)* |  |
| **Animal contact** | 1.8 (0.6,4.07) | 1.29 (0.43,2.93) | -1.080 (-1.105,-1.056)* | 0 (0,0.01) | 0 (0,0) | -1.089 (-1.130,-1.048)* |  |
| **Conflict and terrorism** | 2.85 (1.16,6.52) | 1.75 (0.7,4.01) | -0.941 (-6.089,4.490) | 0.1 (0.03,0.26) | 0.15 (0.05,0.39) | 1.387 (1.246,1.529)* |  |
| **Road injuries** | 4.85 (2.43,9.35) | 3.21 (1.63,6.08) | -1.329 (-1.360,-1.298)* | 0.29 (0.15,0.51) | 0.19 (0.1,0.33) | -1.425 (-1.456,-1.395)* |  |
| **Exposure to mechanical forces** | 4.95 (1.78,10.63) | 3.49 (1.26,7.53) | -1.122 (-1.155,-1.089)* | 0.09 (0.05,0.17) | 0.06 (0.03,0.11) | -1.329 (-1.360,-1.299)* |  |
| **Falls** | 15.09 (7.2,27.92) | 14.77 (7.14,27.67) | -0.053 (-0.170,0.064) | 0.6 (0.31,1.05) | 0.58 (0.3,1) | -0.124 (-0.226,-0.022)* |  |

AAPC: average annual percent change; ASIR: age-standardized incidence rate; ASYR: age-standardized YLD rate; CI: Confidence interval; UI: Uncertainty intervals (*P < 0.05)
